# Supplementary material for: Blobby is a synaptic active zone assembly protein required for memory in Drosophila
Source: Nat Commun. 2025 Jan 2;16:271. doi: 10.1038/s41467-024-55382-9 (PMC11696761; doi:10.1038/s41467-024-55382-9)
Supplement: Supplementary file 1 — Supplementary Information [file 41467_2024_55382_MOESM1_ESM.pdf]

Supplementary Information

Blobby is a synaptic active zone assembly protein required for memory in *Drosophila*

J. Lützkendorf*[1], T. Matkovic-Rachid*[1], S. Liu[2], T. Götz[1], L. Gao[1], O. Turrel[1], M. Maglione[1,3], M. Grieger[1], S. Putignano[1], N. Ramesh[1], T. Ghelani[1,9], A. Neumann[1], N. Gimber[4], J. Schmoranzer[4], A. Stawrakakis[1], B. Brence[5], D. Baum[5], Kai Ludwig[6], M. Heine[7], T. Mielke[8], F. Liu[9], A.M. Walter[9,10], M.C. Wahl[2], S.J. Sigrist[†1,11]

Affiliations:

[1]Freie Universität Berlin, Institute for Biology and Genetics, Berlin, Germany.
[2]Freie Universität Berlin, Institute of Chemistry and Biochemistry/Structural Biochemistry, Berlin, Germany.
[3]Freie Universität Berlin, Institute for Chemistry and Biochemistry, SupraFAB, Berlin, Germany.
[4]Charité- Universitätsmedizin, Advanced Medical Bioimaging Core Facility, Berlin, Germany.
[5]Zuse Institute Berlin, Department of Visual and Data-Centric Computing, Berlin, Germany.
[6] Freie Universität Berlin, Institut für Chemie and Biochemie, Forschungszentrum für Elektronenmikroskopie, Berlin, Germany.
[7]Institute of Developmental Biology and Neurobiology, Johannes Gutenberg University Mainz, Mainz, Germany.
[8]Max Planck Institute for Molecular Genetics, Berlin, Microscopy and Cryo-Electron Microscopy Service Group, Berlin, Germany.
[9]Leibniz-Forschungsinstitut für Molekulare Pharmakologie, Campus Berlin-Buch, Berlin, Germany.
[10]University of Copenhagen, Department of Neuroscience, Copenhagen, Denmark.
[11]Charité Universitätsmedizin, NeuroCure Cluster of Excellence, Charitéplatz, Berlin, Germany

*J. Lützkendorf and T. Matkovic-Rachid contributed equally to this work.

†Corresponding author. Email: stephan.sigrist@fu-berlin.de

The PDF file includes:

Supplementary Figures S1-S7

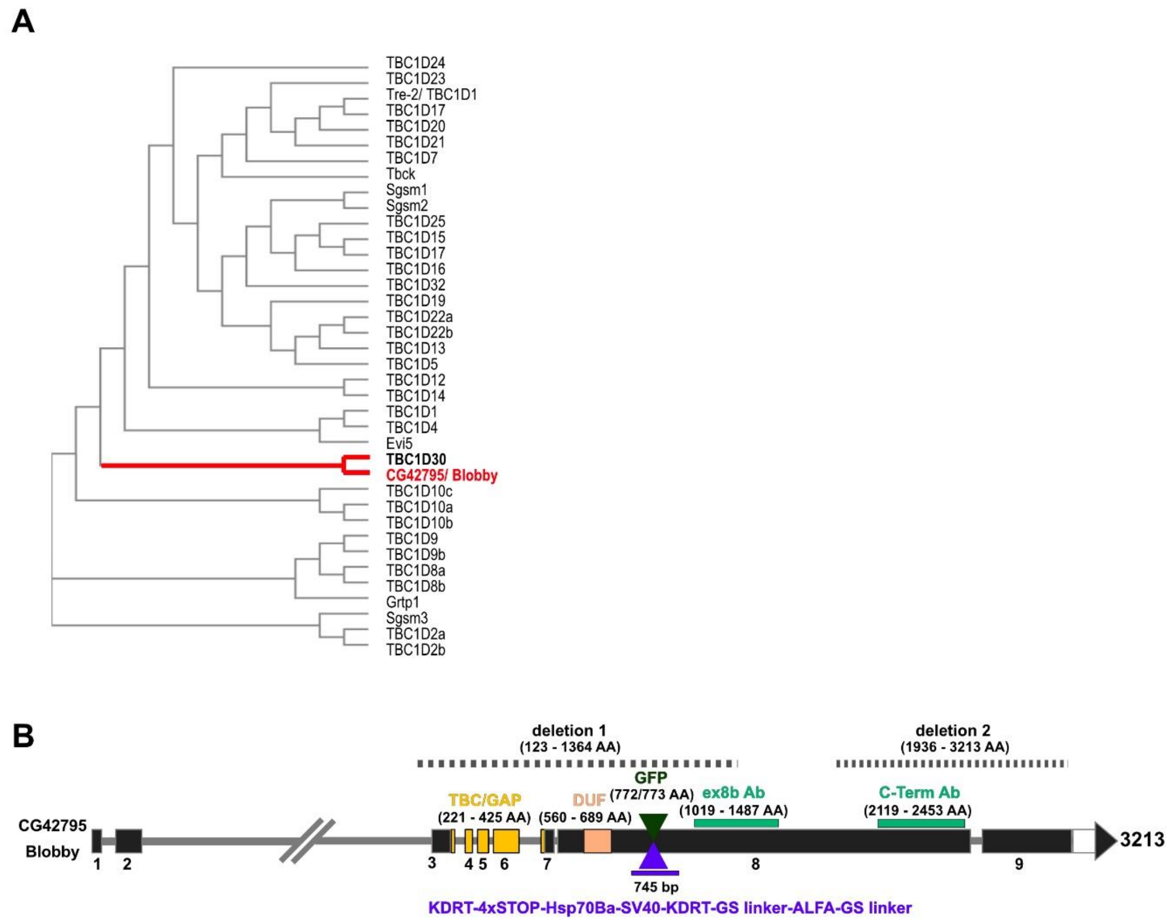

Supplementary Figure 1 | The novel AZ scaffold protein Blobby: evolutionary relations and locus organization.

A) Dendrogram analysis with all predicted human Tre-2, Bub2 and Cdc16 (TBC) containing proteins in comparison with *Drosophila* Blobby. B) Overview map of the blobby gene locus: Tre-2, Bub2 and Cdc16 (TBC) domain (yellow), domain of unknown function (DUF, apricot), indicated antibodies (light green, ex8b and C-term), position of GFP insertion (dark green), position of KDRT-STOP-KDRT-ALFA cassette insertion (purple). For generation of *blobby*^{Null} two parts of the locus were deleted (dotted line; deletion 1 and 2) by using the CRISPR/Cas9 system.

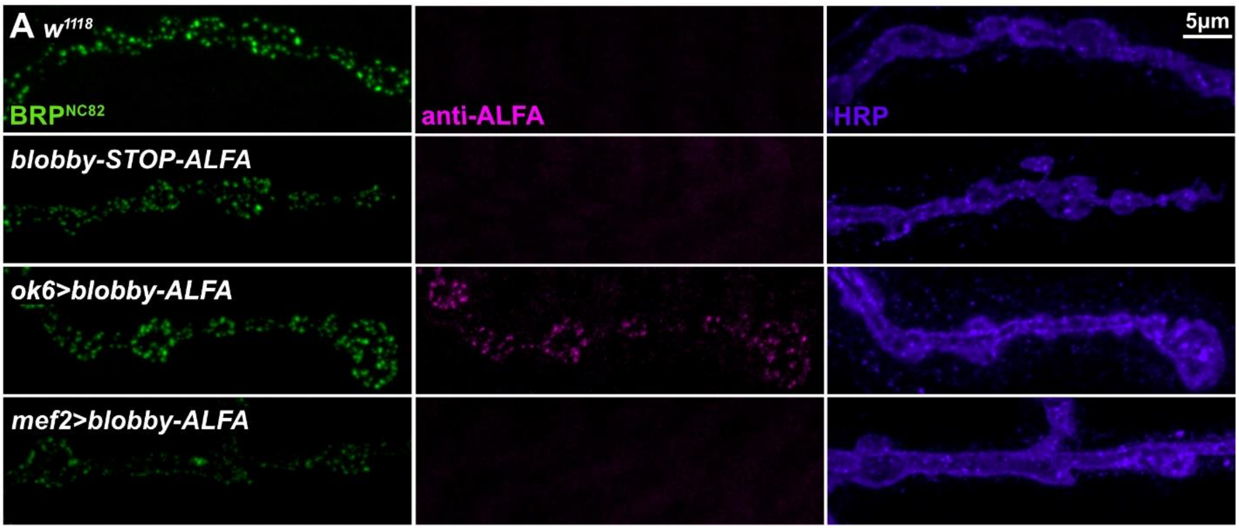

Supplementary Figure 2 | Blobby AZ label derives from the presynaptic motoneuron.

A) Confocal images depicting muscle 4 NMJs from third instar larvae of w^{1118} labelled with the indicated antibodies. *blobby-STOP-ALFA* does not show Blobby expression. Re-expression of Blobby could only be re-established in the motoneuron (with ok6-Gal4) and not in the muscle (with *mef2-Gal4*). Further details are provided in the main text.

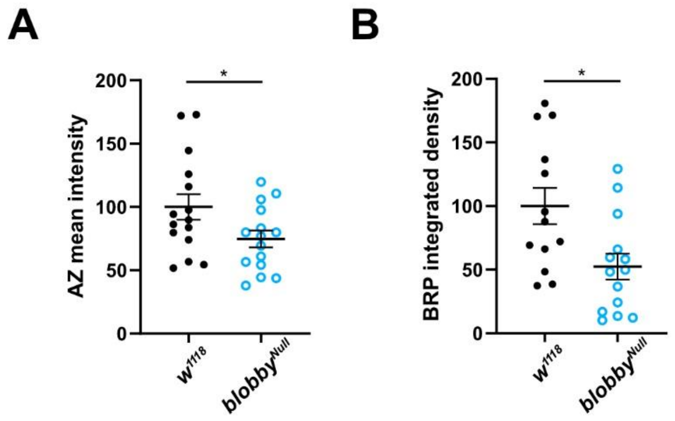

Supplementary Figure 3 | Quantification of BRP levels at *blobby*^{Null} AZs via confocal microscopy.

A) Quantification of BRP^{NC82} mean pixel intensity in *blobby*^{Null} normalized to control (*w*^{1118} 100.00 % ± 3.80, n=32; *blobby*^{Null} 5 72.67 % ± 3.00 n=34) and B) BRP integrated density (#BRP spots * spot area* BRP mean intensity/ NMJ area): (*w*^{1118} 100.00 % ± 7.12, n=25; *blobby*^{Null} 67.65 % ± 7.60, n=29). Graphs show mean ± SEM. n represents the number of NMJs from 5 animals per analyzed genotype. A Kolmogorov-Smirnov test was applied. *p < 0.05; **p < 0.01; ****p < 0.0001. Source data are provided as a Source Data file.

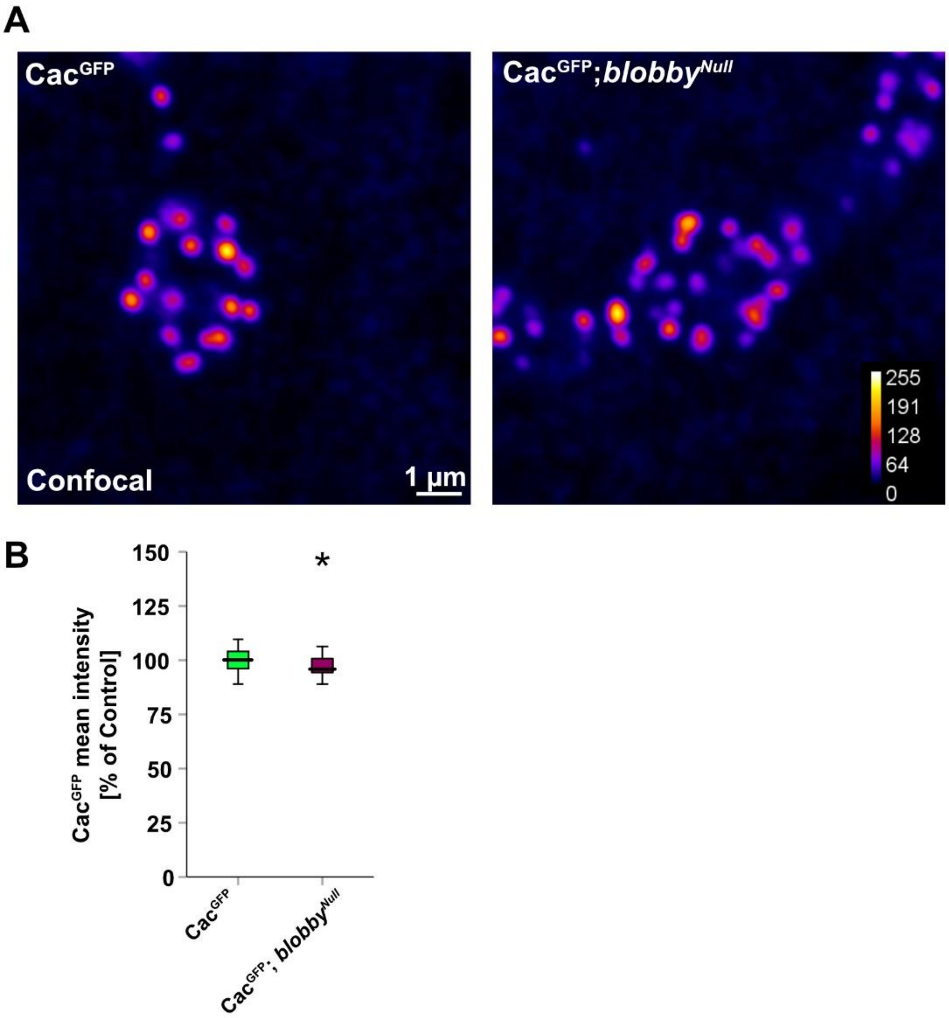

Supplementary Figure 4 | Quantification of Cac^{GFP} levels at *blobby*^{Null} AZs via confocal microscopy.

A) Confocal images of Cac^{GFP} in control and Cac^{GFP};*blobby*^{Null} NMJs. B) Quantification of mean Cac^{GFP} intensity levels in controls and Cac^{GFP};*blobby*^{Null} boutons. Cac^{GFP} 100.00 % ± 0.77, n=45; Cac^{GFP};*blobby*^{Null} 96.97 % ± 0.89, n=30 (n= number of boutons from 3 animals). Graphs shows median, lower and upper quartiles, whiskers represent min/max scores. mean±SEM values. Mann Whitney U Test was performed, ns=not significant. Two-tailed independent Student´s t-Test was performed *p < 0.05. Source data are provided as a Source Data file.

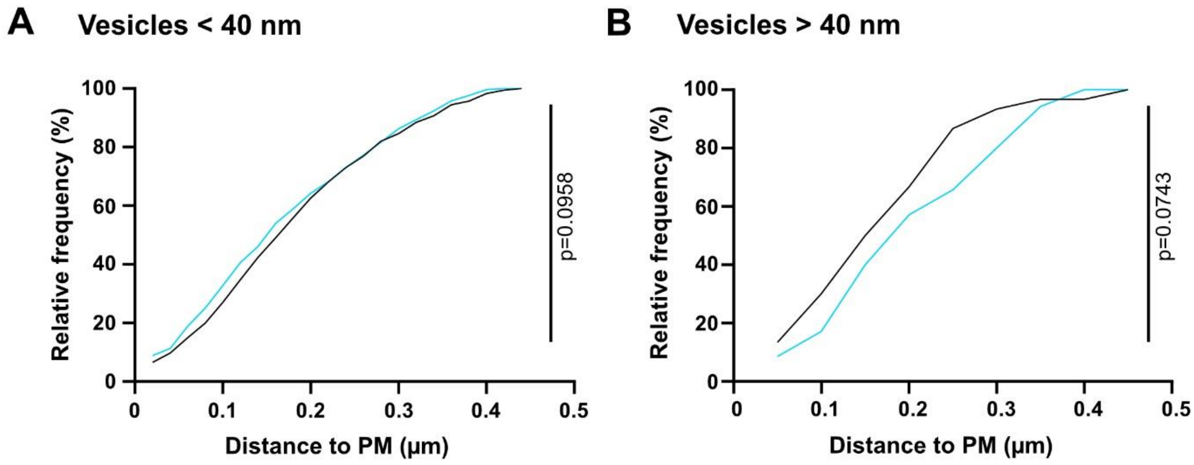

Supplementary Figure 5 | Plasma membrane distance analysis of vesicle profiles smaller or larger than 40 nm vesicle types in *blobby*^{Null} mutants .

A-B) Histogram of distance from plasma membrane within 450 nm of (A) synaptic vesicles (smaller than < 40nm (*w*^{1118} 0.18 ± 0.004 , n=702; *blobby*^{Null} 0.18 ± 0.004 n=497) and (B) of larger vesicles (bigger than >40nm)) (*w*^{1118} 0.17 ± 0.02 , n=30; *blobby*^{Null} 0.22 ± 0.02 n=35). Source data are provided as a Source Data file.

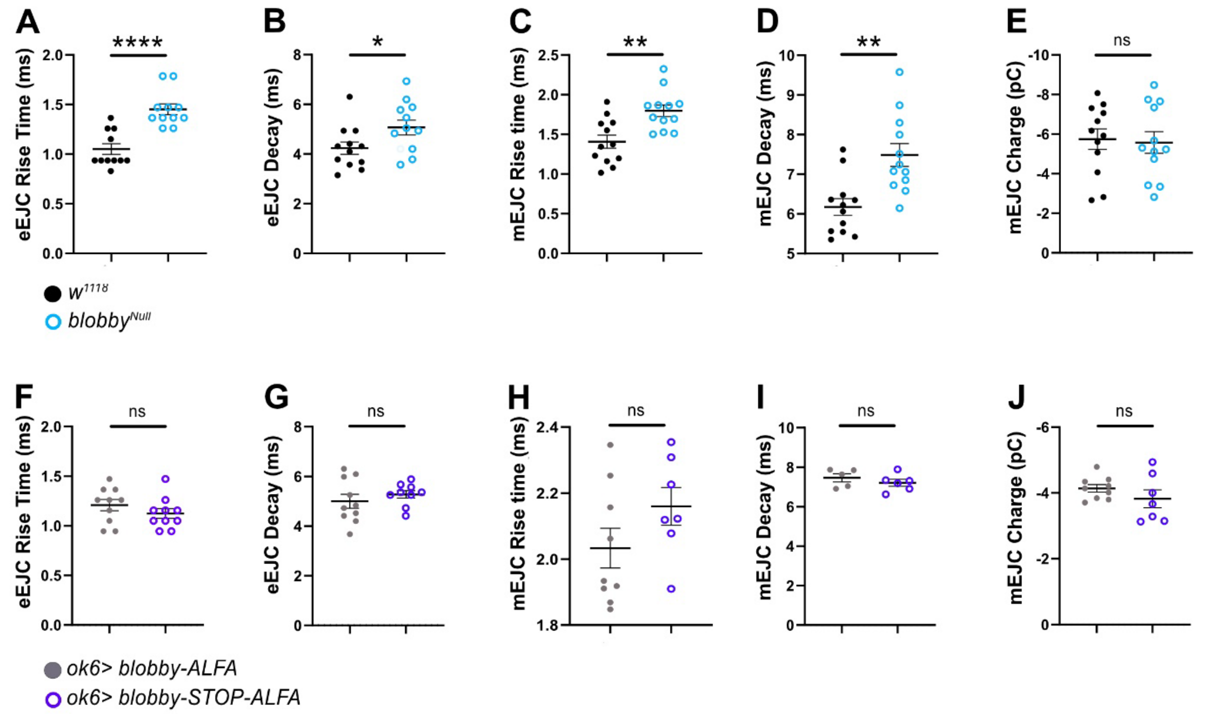

Supplementary Figure 6 | Electrophysiological analysis of *blobby* mutant NMJs.
A-J) Two-electrode voltage clamp electrophysiological recordings comparing third instar larvae NMJs of *blobby*^{Null} animals to controls (A-E) and *ok6, blobby-STOP-ALFA* to *ok6>blobby-ALFA* (F-J).
A, F) eEJC rise time (*w*^{1118} 1.05 ± 0.05 ms, n=11; *blobby*^{Null} 1.45 ± 0.05 ms, n=11; *ok6>blobby-ALFA* 1.12 ± 0.05 ms, n=10; *ok6, blobby-STOP-ALFA* 1.21 ± 0.07 ms, n=10).
B, G) eEJC decay (*w*^{1118} 4.24 ± 0.25 ms, n=12; *blobby*^{Null} 5.07 ± 0.29 ms, n=12; *ok6>blobby-ALFA* -93.42 ± 6.13 nA, n=10; *ok6, blobby-STOP-ALFA* -45.73 ± 3.40 nA, n=10).
C, H) mEJC rise time (*w*^{1118} 1.41 ± 0.08 ms, n=12; *blobby*^{Null} 1.79 ± 0.07 ms, n=12; *ok6>blobby-ALFA* 5.28 ± 0.15 ms, n=9; *ok6, blobby-STOP-ALFA* 5.00 ± 0.28 ms, n=10).
D, I) mEJC decay (*w*^{1118} 6.17 ± 0.21, n=12 ms; *blobby*^{Null} 7.48 ± 0.29 ms, n=12; *ok6>blobby-ALFA* 7.21 ± 0.17 ms, n=6; *ok6, blobby-STOP-ALFA* 7.46 ± 0.20 ms, n=5).
E, J) mEJC charge (*w*^{1118} -5.74 ± 0.52 pC, n=12; *blobby*^{Null} -5.57 ± 0.54 pC, n=12; *ok6>blobby-ALFA* -3.82 ± 0.27 pC, n=7; *ok6, blobby-STOP-ALFA* -4.14 ± 0.11 pC, n=9). Graphs show mean ± SEM. An unpaired t-test was applied, *p < 0.05; **p<0.01; ****p < 0.0001 ns = not significant. n represents a single cell. Four to six animals are analyzed with one or two cells/animal. Source data are provided as a Source Data file.

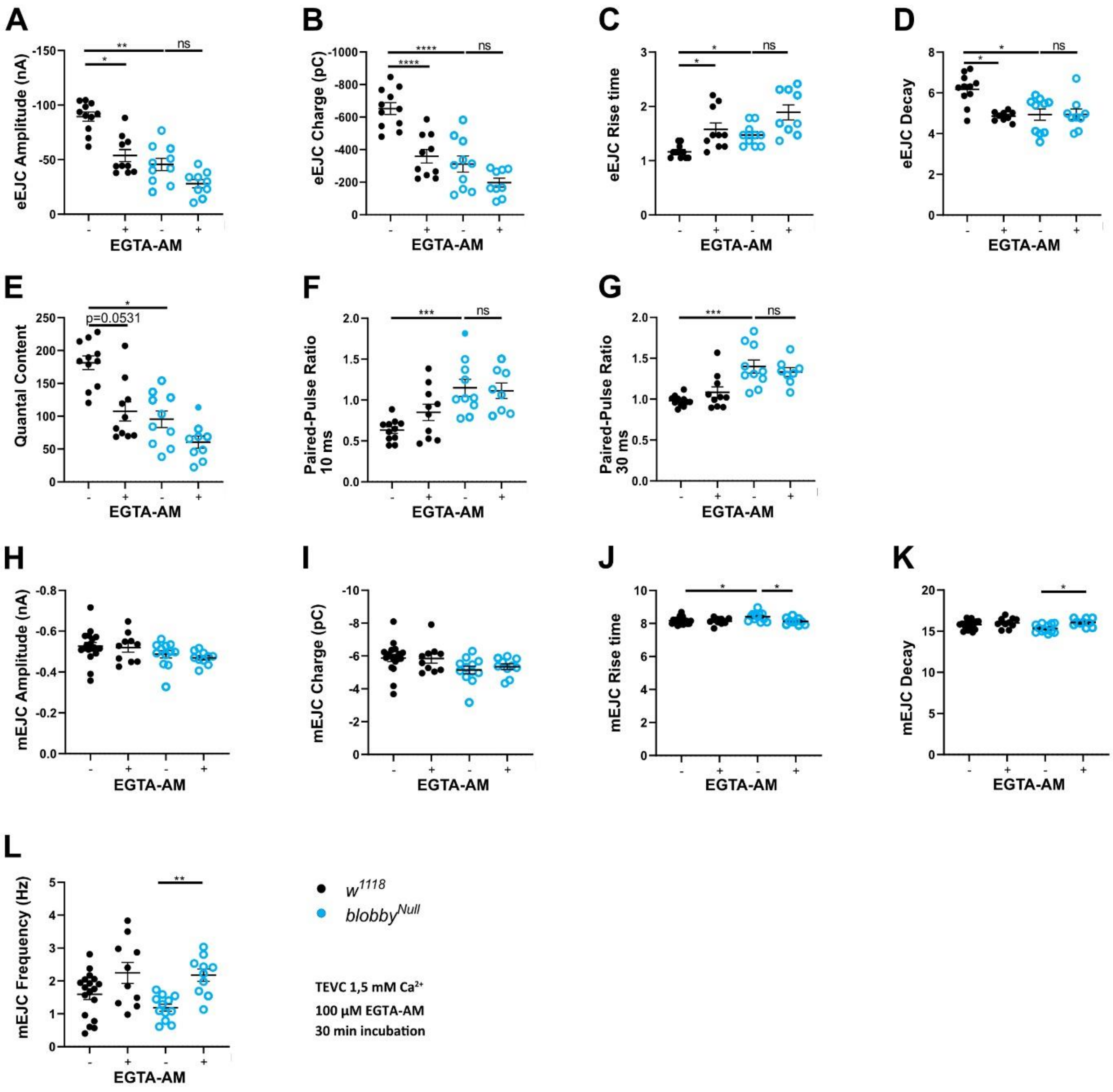

Supplementary Figure 7 | EGTA Electrophysiological analysis of blobby null mutants.

A-L) Two-electrode voltage clamp electrophysiological recordings comparing third instar larvae NMJs of *blobby*^{Null} animals to controls.
A) eEJC amplitude (*w*^{1118} -EGTA -89.48 ± 4.23 nA, n=11; *w*^{1118} +EGTA -53.81 ± 5.53 nA, n=10; *blobby*^{Null} -EGTA -45.66 ± 5.55 nA, n=10; *blobby*^{Null} +EGTA -28.02 ± 3.84 nA, n=9)
B) eEJC charge (*w*^{1118} -EGTA -652.00 ± 36.47 pC, n=11; *w*^{1118} +EGTA -359.10 ± 41.46 pC, n=10; *blobby*^{Null} -EGTA -311.10 ± 49.72 pC, n=10; *blobby*^{Null} +EGTA -197.00 ± 27.27 pC, n=9).
C) eEJC rise time (*w*^{1118} *18* -EGTA 1.17 ± 0.04 ms, n=11; *w*^{1118} +EGTA 1.58 ± 0.12 ms, n=9; *blobby*^{Null} -EGTA 1.47 ± 0.06 ms, n=10; *blobby*^{Null} +EGTA 1.89 ± 0.14 nA, n=9).
D) eEJC decay (*w*^{1118} -EGTA 6.18 ± 0.23 ms, n=11; *w*^{1118} +EGTA 4.86 ± 0.08 ms, n=9; *blobby*^{Null} -EGTA 4.94 ± 0.28 ms, n=10; *blobby*^{Null} +EGTA 4.95 ± 0.26 nA, n=9).
E) quantal content (*w*^{1118} -EGTA 181.30 ± 10.49, n=11; *w*^{1118} +EGTA 107.40 ± 14.61, n=9; *blobby*^{Null} -EGTA 95.42 ± 12.64, n=10; *blobby*^{Null} +EGTA 60.51 ± 9.18, n=9).
F) paired pulse ratio 10 ms (*w*^{1118} -EGTA 0.63 ± 0.04, n=11; *w*^{1118} +EGTA 0.85 ± 0.10, n=9; *blobby*^{Null} -EGTA 1.15 ± 0.10, n=10; *blobby*^{Null} +EGTA 1.11 ± 0.09, n=8).
G) paired pulse ratio 30 ms (*w*^{1118} -EGTA 0.98 ± 0.02, n=11; *w*^{1118} +EGTA 1.09 ± 0.07, n=10; *blobby*^{Null} -EGTA 1.34 ± 0.08, n=10; *blobby*^{Null} +EGTA 1.33 ± 0.05, n=8).
H) mEJC amplitude (*w*^{1118} -EGTA -0.53 ± 40.02 nA, n=18; *w*^{1118} +EGTA -0.52 ± 0.02 nA, n=10; *blobby*^{Null} -EGTA -0.48 ± 0.02 nA, n=12; *blobby*^{Null} +EGTA -0.47 ± 0.01 nA, n=10)
I) mEJC charge (*w*^{1118} -EGTA -5.88 ± 0.02 pC, n=18; *w*^{1118} +EGTA -5.84 ± 0.28 pC, n=10; *blobby*^{Null} -EGTA -5.14 ± 0.23 pC, n=12; *blobby*^{Null} +EGTA -5.35 ± 0.17 pC, n=10).

J) mEJC rise time (*w*^{1118} -EGTA 8.17 ± 0.06 ms, n=18; *w*^{1118} +EGTA 8.13 ± 0.06 ms, n=10; *blobby*^{Null} -EGTA 8.41 ± 0.07 ms, n=12; *blobby*^{Null} +EGTA 8.13 ± 0.06 nA, n=10).
K) mEJC decay (*w*^{1118} -EGTA 15.81 ± 0.13 ms, n=18; *w*^{1118} + EGTA 16.02 ± 0.19 ms, n=10; *blobby*^{Null} -EGTA 15.34 ± 0.15 ms, n=12; *blobby*^{Null} + EGTA 16.05 ± 0.14 nA, n=10).
L) mEJC frequency (*w*^{1118} -EGTA 1.59 ± 0.16 Hz, n=18; *w*^{1118} + EGTA 2.24 ± 0.32 Hz, n=10; *blobby*^{Null} -EGTA 1.19 ± 0.11 Hz, n=12; *blobby*^{Null} +EGTA 2.18 ± 0.19 Hz, n=10). Graphs show mean ± SEM. An ordinary one-way ANOVA test was applied, *p < 0.05; **p<0.01; ***p<0.001 ****p < 0.0001 ns = not significant. n represents a single cell. Four to six animals are analyzed with one or two cells/animal. Source data are provided as a Source Data file.

Supplementary Table 1 | List of proteins identified by BRP co-immunoprecipitation from isolated and purified synaptosome *Drosophila* extracts. Data show the average log2 fold-change of identified proteins and the corresponding p-values of a two-sided t-test without multiple hypothesis correction. The cut-offs were set manually at $p < 0.05$ and log2 fold-change > 2. Proteins passing these cut-offs are listed with UniProt IDs and gene names.

UniProt Protein IDs	Gene names	p-value	fold change
**Q9VH10;A0A0B4JCW7;A0A0B4LGZ2;A0A0B4LH08;A0A0B4KG40;A0A0B4LH38**	CG42795/ Blobby	5,49	12,15
**A0A0B4K843**	brp-I	4,01	10,27
**A0A0B4LFH0;A0A0B4KG54**	Mtap	7,23	10,10
**E1JIQ1;A0A0B4LHB7;A0A0B4LHB9;A0A0B4KHS0;E1JIQ2;Q0IGX3**	GluClalpha	6,26	9,51
**Q9VXL1;X2JE21**	mmd	3,47	8,69
**A0A0B4K866**	stj	4,61	8,58
**A0A0S0X7W8;A0A0S0X0Z7;M9PGE0;M9NEN6**	CG3638	7,23	8,55
**Q7K4Y8**	CG1909	7,75	8,51
**H9XVP2;Q8IM86;D0Z730;C3KGK3**	unc-13B	6,56	8,41
**B7Z0R2;A0A0B4KHD2;Q9VAS8**	spg	6,96	8,24
**M9PDH4;M9NFQ4;M9PG86;M9NEP4;M9PIP8;M9PGF5;C7LAE9;M9NGH0;M9PGM6;M9NE32;C7LAF2**	CG32809-RB;CG32809-RD	7,30	8,24
**A1Z9M5;Q95TJ2**	CG30069	6,83	8,22
**A8DY77;Q7K304;A0A0B4KFF6;F3YD50;Q9U787**	Drep2	5,08	8,17
**Q9VFS1**	Adgf-D	5,25	8,12
**A8JNL5**	sif	8,40	8,09
**Q9W483**	CG15772	7,05	8,04
**A0A0B4KF38;Q1EC18**	brp	7,05	8,03
**Q9VVY7;Q6NR14;R9PY60**	CG32206-RB;CG32206	6,42	8,01
**M9PHB3;A0A140B9G0**	Sxl	7,09	7,64
**A0A0B4LJ24;A0A0B4K7G3;A0A0B4K6W5;I0C0N0;A0A0B4K813;A0A0B4K7S3**	RhoGAP100F	4,93	7,42
**A8JQT5;A4IJ62;Q9VN68;A2VEE0**	Cdep-PG	6,23	7,31
**Q6IWN7;X2JDW0;Q9W581;Q8MM14;E1JJC6;O76903;Q8MX77;Q059A8**	Nmdar2;CG33513-RC	4,57	7,31

**Q9VPA1;M9PG87;Q95U78**	CG3116	5,29	7,21
**A8DYL2;Q86P20**	CG13497	4,41	7,17
**Q8T6L6;Q8T6L7;Q4AB27;Q961G2;Q4AB24**	Myo95E	5,53	6,97
**M9PER3**	CG13293	5,67	6,95
**A8JR71;A8JR72**	CASK	4,57	6,90
**E1JIV7;A0A0B4KHT2**	slo	4,65	6,81
**Q25B55**	brp	7,74	6,61
**A0A0B4KG15**	aPKC	6,72	6,55
**Q9VG01;A1A735**	CG46280	4,13	6,49
**E1JIS7**	CASK	7,26	6,42
**Q3KN41;A0A0B4KGL0;A0A0B4KGQ3;Q9VCZ9;A0A0B4KH61;Q8SZX4**	Nrx-1	4,89	6,27
**M9PHI5;A0A0S0WGZ0**	sif	6,94	6,27
**M9PFN1**	rept	4,24	6,27
**Q960X9**	Lon	4,90	6,22
**M9PBG8;Q9W0S2;B7Z0H3;Q8IRJ2;Q8IRJ3;Q8IRJ4;Q9W0R9;Q9W0S0;Q6NN79;Q8MSB6;A1L4U0**	RhoGEF3	5,14	6,15
**A0A0B4KHE6;A0A0B4KGU3**	nAChRalpha1	5,06	6,09
**B7Z048;Q9VU98;M9PFJ0;M9PFF3;M9PI53**	dysc	6,96	6,08
**Q9VE20;A0A0B4K760;A0A0B4K6C8;A0A0B4K686;Q961I3;B3DNL6**	CT33918	3,27	6,03
**A8DYB0;A2VEG7;Q95TG7**	CG13185	6,28	5,99
**Q9VP80;Q5U159;Q95U36;Q6U9V3;E1JI59;Q7KTX2;Q5U112**	siz	5,88	5,98
**E0R955;Q6NKM7**	hig-RC	5,87	5,95
**M9PC26;M9PEE5;M9PBX4;M9PB25;Q9VQU8;C9QP05;Q8IQ16;Q8MZ53;M9PBX6**	CG31772	4,83	5,93
**M9PD84;M9ND67;Q7KTE7;M9PFL4;Q7KTE8;M9PCW6**	Ca-beta	5,55	5,93
**A4V133;Q52KB0**	CaMKII	6,31	5,91
**E8NHB5**	nAcRbeta-64B-RA	4,77	5,91
**Q9VKB2;Q8MS15**	Ced-12	4,36	5,89

**X2JIM3;X2JCS0;Q9W406;A8JUZ7**	CG15894	6,56	5,89
**X2JAF1**	lva	4,94	5,82
**A8JNX5;Q9NFR5;M9PDC9**	nAChRalpha4	5,38	5,73
**X2JAM1;Q9V9Q7;E1JHP3;Q0E8N3;Q9V9Q9;X2JEX4;C0PTX3;A1A6V1;Q8MRW9**	nolo;CG31619-RB;CG31619	6,78	5,72
**Q86P77;Q9VDE9;Q7YU00**	RhoGAP93B	5,48	5,69
**A0A0B4K6B1;A0A0B4K6M2;V5M2P5;A0A0B4K742;V5M054**	Rim	4,96	5,68
**Q86MN7;Q9VWI9;A4GXC7;E1JJR2;C5WLN7;Q8T5F4;C6SV01**	nAChRalpha7;nAcRalpha18C;gfA-RA	5,87	5,64
**Q9VM93;M9PCR7;M9PCD4;M9PC73**	Liprin-alpha	5,73	5,62
**Q9W1F1;C6TP52;A0A0C4DHD7;Q29QH9;Q9U523**	alpha-catenin-related;alpha-catenin-related-RA	5,00	5,60
**T2GFI6;Q0E993;Q960E6**	Aats-val-RA;Aats-val	5,47	5,47
**H5V864;D1Z359;M9NEY5;M9NEY3;M9NFI9;M9NDX8;Q8MSU5;M9NFI2;M9ND28;Q9GQA3;D3DMW9**	Spn;Spn-RA	4,46	5,40
**Q8T7V5;Q7KT97;Q8T5F5**	nAChRalpha5	4,40	5,37
**Q7PLI0;Q9NHP1;Q8T0H3;B5X0M8**	p120ctn	3,54	5,31
**Q1LZ41;F0JAM6;Q8IRS0;E1JJE4;X2JCK2;Q9W4F5;Q24495;Q8SXB2**	Ptp4E;Ptp4E-RC	5,92	5,31
**B6IDW7**	Act88F-RA	5,30	5,28
**M9PCI8;E1JHT0;A0A0S0WIF3;E1JHS9;A0A0S0WJJ4;C0PTW0**	tutl;tutl-RD	5,70	5,26
**X2JD88;M9PE96**	CG15207	4,33	5,26
**Q9VPR5**	Sf3b1	6,21	5,26
**Q0E8S7**	homer	3,72	5,23
**Q9VZI4;Q8IRC2;Q8I0Q7;E1JIC5;F0JAM2;Q95U18;E1JIC6;A2VEF8;C4XVI4**	CG14995-RC;CG14995-RD	7,92	5,22
**Q8T9G9;Q9VAU5;Q8T4F8**	CG10011	5,81	5,13
**Q9VKR7;Q95TX4**	CG7300	6,18	5,13
**Q9VBY7;Q8IMT8;Q6NL82**	veli	7,76	5,11
**Q8IM87;Q8SX82;Q8T049;Q9U4K9**	unc-13A	4,80	5,08
**Q8IQQ0;Q8IGI6**	Nc73EF	6,10	5,08
**U3PXB4;B7FNQ8**	Sra-1-RA	3,47	5,06

**M9PGF7**	Ten-m	4,18	5,03
**Q9VNF8;A0A0B4K6T4;A0A0B4K5Z8;Q8IGT0;Q9NFN9;Q95TD2**	Sec23	2,62	5,01
**Q9VJ94;Q1WWB2;Q9VJ91**	Dipeptidase	4,84	4,99
**A0A0C4DHG2;A0A0C4DHG6**	Gel	3,77	4,97
**Q95SQ6;Q9UAI4;O76137;A0A0B4KFZ4**	TM4SF	7,78	4,97
**A0A0B4KFR2;A0A0B4KEV7;A0A0B4KG20;A0A0B4KER3;A0A0B4LFI1;A0A0B4LGK0;A0A0B4LFG4;A0A0B4KF73;A0A0B4LF79;A8DYE9;A0A0C4DHC0;Q29QR2;Q1EC92;A8DYE4;A0A0B4LFF3;Q8SWT1;A1ZA11**	Stacl	5,36	4,95
**Q9VI64**	CG10098	5,60	4,94
**Q9V3P3**	REG	5,40	4,89
**C7LA93;E1JHK3**	dl	5,99	4,89
**A0A0B4KF89;Q2XXX2;B4YXI0;B4YXH7;B4YXH6;A0A0B4KFS9**	gprs	5,33	4,88
**M9PCE5;Q9VU94;Q9U5D0;Q8MS79;Q8SXA9**	Hml	2,91	4,88
**A0A0B4LFA9;A1Z9K8;A0A0B4LF25;A0A0B4K7Z0;F0JAG9;Q961U4**	Prosap;Prosap-RB	5,33	4,85
**H9XVM6;H9XVM7;Q95RW2;D3DMC5;X2JD84;E8NH51;X2JCS2**	Crk	4,57	4,84
**H5V8D0;C1C3C5;X2J5L4;F2FB81;M9NCT2**	SCAR;SCAR-RA	5,98	4,82
**A8DYB7;Q0E9B2;A1Z904;E8NH80;A1Z906;E2QCB7;A1Z907;A0A0B4K6Z2;Q9GT70;Q6NN51;Q95SU4**	Dyb;Dyb-RC	4,27	4,79
**X2JCN4**	kug	5,94	4,79
**Q8SYF0;Q8IPC5;Q8IPC3**	CYLD	5,06	4,77
**M9PHJ5;M9PJM7;Q6NNU5;D6W4T4;D3DMP9**	g;g-RB	4,33	4,77
**Q95U11;Q9VME3;T1W2R4;Q8IGB8**	CG9507;CG9507-RA	4,82	4,76
**A0A0B4KHS9;Q95V52;A8QI94;Q9VQL9;Q6NNT3**	Pp1alpha-96A	4,61	4,75
**A0A0B4KI34;A0A0B4KHG1**	cindr	2,55	4,73
**Q9BML6;Q8IN24;Q9Y133;A0A0B4LHR4**	GABA-B-R2	3,03	4,72
**A8JR03;A8JR02;A0A0B4KGM4;A0A0B4KH30;A8JR00;A8JR01;A0A0C4DHA9;A8MZR2**	kramer	5,09	4,71
**A0A0B4KG52**	HtrA2	5,47	4,69

**A1Z6P8;A0A0B4KF54;Q7K3Z6**	Epac	4,80	4,68
**Q9VML2;Q8MRY5**	bchs	2,66	4,67
**M9NG38;M9NEZ2**	sdt	7,39	4,66
**A0A0H4XWW0;A0A0B4KGU4;M9W6J0;Q9NBW6**	bel;bel-RB	2,95	4,65
**Q9VRP4;Q29QT3**	Ppat-Dpck	1,65	4,65
**A0A0C5KMR0;M9PHG0;M9PHY4;M9PHV3;M9PJQ3;D1Z398**	RhoGAPp190-RB;RhoGAPp190	3,18	4,61
**A0A0B4KH51;Q9Y0S9;A0A0B4K774**	Abi	3,63	4,60
**M9PGT0;M9PDW8;X2JDY1;A8JV00;M9PH75;A8JUZ9;X2JIN0;M9MSA5;Q961G7**	CG34417	3,83	4,59
**D5AEP6**	AnnX-RA	2,27	4,58
**Q9VHC7;Q8MSI9;A0A0B4K6Z1**	rump	4,33	4,58
**A0A0B4KEG9;E1JH11;A0A0B4K6T3;F7VJV3;A0A0B4KEF5;A0A0B4K7U8**	Cirl;Cirl-RF	4,19	4,58
**Q9W3K6**	CG2258	6,88	4,55
**Q9V3V9**	EndoGI	3,77	4,55
**Q7KUA4;Q7KJV6;Q9VSD9**	Uba2	4,12	4,52
**Q7KK29;Q9VEN9;Q6NN42**	Patr-1	5,18	4,51
**I1WYI4;G7H851;X2JBG9**	alpha-Cat-RA	2,63	4,51
**A4V1B2**	Patj	6,03	4,51
**Q27IR0;Q7KY08;Q32KD4;Q960T1;Q8T059**	AGO1	3,71	4,50
**O97417;Q94882**	didum	4,01	4,48
**Q9W437;C3KKC3**	raptor;raptor-RA	6,43	4,47
**Q8IGR6;Q8I935;I0DHK9;M9PBR6;Q9VRR4;Q8IHC4;Q8I934;D3DMM9;Q8I0D5;M9NF21**	Vap-33B;CG33523-RD	1,84	4,45
**M9PFK6**	Fbp1	4,06	4,43
**Q9VYT3;Q5U158;Q8SZT9**	CG2025	3,17	4,42
**Q9W5W8**	CG9577	3,94	4,39
**Q9XYQ9;Q9W3I9;Q9UAC4**	Traf6	3,40	4,39
**A0A0B4K6D2;E8NH46**	CG16817-RA	4,76	4,38
**Q9VXK9**	CG9170	3,80	4,38

**M9PE32;M9PDV6;Q9VZY3;G4LU40;L7Z8N6;Q86NW3;F9W330**	Fife;	4,29	4,36
**X2JL49**	CG7332	4,60	4,34
**A2RVG6;M9PCH6;Q9VUH1**	ome	2,76	4,34
**A1Z8K9**	Sod3	5,33	4,33
**Q9VMC7;A8DYW1**	CG9545	7,29	4,32
**A8JNK1;M9PHB1;Q8IRB9;M9PEK8;M9PBN6;F2FB68;E6PBW7;Q9VZI0;Q9I7T2;Q8IRC1**	ens;ens-RA	5,96	4,32
**Q8IRQ5;Q9W3X6;Q8SXM1;Q9W3X5;B4YWB2;B4YWA6;B4YWC0**	l(1)G0255	2,33	4,32
**Q9VQQ6;X2J8I7**	Snx1	2,87	4,31
**Q4ABE7;Q4ABE8;Q4ABF0;Q4ABE9**	CadN	5,35	4,30
**Q59E51;Q59E50;Q59E53;Q59E52;M9NDV1;M9NEE2;M9NGZ9;M9PHD4;Q8MRR4;C3KGQ4**	cac	4,04	4,29
**Q7KJA9;Q9Y148;Q95RP9**	sxc	2,59	4,28
**M9PEY7;M9PER1;M9PHS3;Q9VSF0**	Atg18a	6,20	4,28
**A1Z6N4**	Tdc2	4,77	4,26
**A8DY43;A0JQ58;Q1RKV1;A9UND7**	CG13556	2,66	4,24
**E1JHD6;Q8IGX1**	bsk	5,34	4,24
**A0A0B4JDC8;Q9VFD3;B9ER11**	CG42788	3,96	4,22
**M9NDR2;M9PDL9;M9PDB8;D3DMV8**	BicD	5,60	4,21
**Q9VFP6;O76455**	Ipp	2,99	4,21
**M9NEI4;M9NGC6;M9MS50;M9NDZ1;M9PIK8;M9NDF9;M9PDA8;Q8SWX7**	srpk79D	5,32	4,17
**X2J5G6**	RpL7	2,65	4,16
**Q7K3V6**	mEFTu2	3,67	4,16
**A8Y560**	RpL15	4,21	4,14
**A0A0B4KFK3**	SLO2	5,15	4,13
**A5HBQ4**	Nrx-1	4,25	4,10
**Q8SX06**	nkt	3,23	4,08
**A8JMD5;M9PF67;C6TP33;Q1EC15;A1A6X8**	CG34356-RC;CG12524	5,34	4,08
**A8JNN5;Q9VSI2;Q8MT33;A2RVI6;A2RVI2**	GAPcenA	2,59	4,07

**X2JCS6**	RpL7A	2,76	4,07
**Q9VQL6;M9PB21;Q8MR70**	v(2)k05816	1,35	4,06
**B7YZS3**	tamo	3,93	4,03
**Q9VDC9;Q961J2**	Oga	3,82	4,03
**Q9VCW6**	Gclm	2,69	4,03
**M9PIS3;A0A0F6T1J9;K7ZI00**	Pgd	8,45	4,02
**A0A0B4K728;A0A0B4LIC0;Q9VEF7;A0A0B4 KHP1;A0A0B4K6L3**	CG14318	3,85	4,01
**Q9W1I8**	Snap29	2,23	4,01
**M9PHL3;M9PJN1;M9NF51;M9PH52;M9PEL4**	rut	3,71	4,01
**A1Z992;U3NIM6;Q8T0H4;B5RJS1**	AGBE	4,29	4,00
**Q7K3W2**	CG8728-RA	4,01	4,00
**Q4V4E7**	CG31119	3,41	3,99
**M9PCC1;M9PB67;B4YX01;B4YX00;B4YWZ6; F6J206;F6J1Z8**	Rack1	1,66	3,96
**C4IY22**	CG42236-RD	5,39	3,95
**E0WN35;Q9W3G6;O18394;O44202**	nicra3;nAChRalpha3	5,13	3,94
**A0A0B4LID2;Q06AJ1;E1JIP9;A0A0B4LHB4; Q9VDY5;Q6NKM8;E1JIQ0**	unc79	3,99	3,94
**Q9VRG8**	CG1486	1,75	3,93
**Q9VWP2;Q8SYA6**	CG7322	3,84	3,92
**Q9VFT4;Q9NH72**	rin	4,04	3,91
**M9PBE5;M9PDS6;M9PDS2;M9PBE4;M9PD86 ;M9PGA0;Q9VIR0;X5CN34;Q058X9**	sick	3,05	3,91
**Q9VKT1;Q9VKS9**	CG17093	3,18	3,89
**Q9W3M7**	mahe	3,51	3,86
**A8JNP1**	Argk	1,68	3,86
**Q9XYZ9;D2NUG2**	GstE12	2,22	3,86
**M9PD95;C1C5B1**	cact	5,47	3,84
**Q9W1R3;Q8T8Q5**	Golgin245	5,08	3,84

**Q7JWX3;Q6V6U1;Q6V6U7;Q6V6U5;Q6V6U9;Q6V6U4;Q6V6U3;Q6V6U2;Q6V6U6;Q6V6V0;Q6V6U8**	nec	3,11	3,82
**M9NDL7;Q8MQS4**	Reps	4,11	3,81
**A1Z6H7;Q6NP18;Q9GPI0**	Gp210;gp210	3,17	3,79
**Q8SYL2;B7Z009**	Shaw	1,70	3,79
**M9NDU6;A4V4C1;C9QPE1;M9NGA3;M9PHA6;Q9NGY7;Q9NGY8**	CkIIbeta	3,43	3,75
**Q7KUW2;Q9VWV5;D0IQG9**	CG32549	3,50	3,75
**M9NEC1;M9PFS5;E1JI13;M9PCU3;M9PI84;M9NDT7;M9NG14;C5WLS8;F3YDL8**	Dab	3,24	3,75
**Q7KV94;M9PE19;Q7KV91;Q7KV95;E4NKM8;Q95TC1;M9ND26**	CG16758	4,22	3,75
**Q7K523;Q9VEZ0;Q0KI67;Q8INC3;Q0KI66;Q8IH49;A0A0B4KGR6;Q8INC1;Q8T6M0;Q8SZW3;A0A0B4JCY5;A4IJ75;O96816**	Mhcl	4,12	3,74
**U3PXA7**	sec8-RA	5,33	3,74
**Q8T0U6;Q8SWU9;H5V895**	flw	5,89	3,73
**M9PDE6;R9PY70;Q9V9N5;M9PDY6;Q9V9N7;M9PBG0;A8DZ25;M9MSJ5;A2VEE9;M9PDL8;A8DZ24**	CG15217	2,07	3,72
**Q9VNI6**	CG31555	2,68	3,71
**Q0E9G4;G3LFI2;Q961L4;G3LFG9**	Drat	4,53	3,70
**Q967T6**	gag	3,52	3,70
**M9PCI5**	CG8475	5,38	3,70
**A0A0B4JD00;A8DY69;A0A0B4JCQ5;Q8SXP0;A0A0B4JD31;E5DK16;A0A0B4JD27;A0A0B4JCS1;F0JAG5;I0E2I4;A2RVH5**	Lpin	3,72	3,70
**E2QD61;A0A0B4K6W3;A0A0B4LHW4;A0A0B4K634;A0A0B4K6B6;A2VEX8**	CG31187	3,95	3,69
**Q8MZI3;Q7KU78;Q8T0I3**	DmRH5	3,90	3,68
**B5RJG2**	dor-RA	3,93	3,68
**Q9W330;Q9GNH8;Q9GQS3;A9YKE5;Q9BK28;B4YWN4;B4YWM6;Q1NZ60**	Hex-A	3,39	3,68
**X2J8Y6**	Acp36DE	3,80	3,67
**M9PHS6;B7Z0D9;Q0E8G9;Q3ZAL9;D3DMK1;Q1RKW0**	Dscam4	4,04	3,66

**X4YX01;Q9VCW7;A0A0B4LHH4;A0A0B4K6D0;D0Z761;A0A0B4K6W6**	wake	3,72	3,66
**A0A1W5Q0S1;E1U3F9;E1U3G3;E1U3F8**	for	2,48	3,66
**Q9VU76;M9PFE5;M9PFI2;Q961X4**	cmb	4,81	3,65
**Q8MRI4**	CG1578	4,94	3,65
**X2JF15;X2JEQ0;X2JBI1;X2JJU0;X2JDH0;Q9I7S4;A2VEW4**	rad	3,17	3,63
**M9PEX9;M9PBV5;M9PEG9;M9PEQ0;M9PHR0;M9PEH2**	syd	3,96	3,63
**Q86DS7;Q53ZT0**	DnaJ-1	4,59	3,63
**Q8T3L2**	vib	1,95	3,62
**Q8IMP0;C5WLQ7;Q8IGZ4**	CG31064-RE	4,27	3,62
**X2JAI2**	Arpc2	3,76	3,62
**Q9VJZ6**	Grx4	3,09	3,61
**Q7KSP6;Q960A9;Q9VGH9;O96610**	Sbf	5,26	3,60
**Q9VS41;Q6NN63**	unc-13-4A	3,96	3,59
**Q8T7S2;Q86MN8;Q9VL79;Q8T7S1;Q8IPE2;Q7KTF9;M9PFD8;Q6NNY5;Q8T7S0;Q7KTF7;Q8T7R9;Q7KTF8**	nAChRalpha6;nAcRalpha-30D	4,03	3,58
**A4V2B8**	CkIIalpha	3,65	3,58
**A0A0S0X7Z4;E1JJQ5**	sh	2,69	3,57
**Q9W003;D6W4L6**	Spn	4,19	3,56
**M9PH69;Q9VXV8;M9NEI3;M9NGG3;Q9U5E2;B8Q7Z3;Q9VXV7**	Cngl	4,03	3,56
**Q9VEK8;Q9U3W5**	sds22	4,23	3,54
**Q9XYX1;M9NEH6;C6TPA9;M9NGF7;Q86NK9;I1V507;C5I7Q8;C5I7G2;C5I7E6;C5I7C2;C5I798;Q8IR38;I1V506;C5I7Q9;C5I7G3;C5I7E7;C5I7C3;C5I799;Q8IR37;I1V508;C5I7R0;C5I7G4;C5I7E8;C5I7C4;C5I7A0;M9PJN5;C5I7H8;C5I7H9;C5I7I0**	HDAC6	4,51	3,53
**Q7K2L7**	CG17765	3,74	3,53
**Q7K8Y3;Q8MPN5;Q8MPN6;Q8MM39;Q9U1I5;Q7K8Y5;Q8MPN7;Q8MPN8;Q8MM49;Q8T0M5**	Spn42Da	3,18	3,53
**Q7K1W5**	CG8187	2,60	3,53

**M9PDZ8**	Ubr3	2,60	3,52
**M9PC85;Q9V3V0**	x16	3,24	3,52
**Q9VE62;Q7KSD3**	fray	4,86	3,50
**A0A0B4K7W3;A0A0B4K7I2;Q7JMZ7;Q6NND8;A0A0B4K7I6;A0A0B4K7B0;A0A0B4K852;A0A0B4KF04;A0A0B4K7H8;A0A0B4K7W7;A0A0C4DHD5;A0A0B4K8A2;A0A0B4K8A1;A0A0B4KEV0;A1ZBN3;Q8IHH3;D5AEJ9;Q0E914;Q86P89;D4G7B7**	sm	2,55	3,50
**M9NFZ9**	ringer	3,80	3,49
**S0AT10;T2FF94**	PPO3-RA;proPO59-RA	4,49	3,49
**Q9W3C3**	CG2004	3,94	3,48
**Q9VZK7;Q8T919**	CG14982	3,69	3,46
**Q9VB22**	pins	4,59	3,46
**Q9VTU9;M9PI33**	CG6910	4,52	3,46
**A0A0B4LFM0**	Cbp53E	3,13	3,45
**A0A1B3Q3R0;A0A0F6PCQ7;G8E4R5**	RpL32	3,72	3,45
**E1JHK2**	Dif	4,58	3,45
**A4V3J6;C8VV67;A4V3J5;E6PBW8;E8NH29**	Hrb98DE;Hrb98DE-RE;Hrb98DE-RC;Hrb98DE-RA	2,21	3,44
**Q9VZ38**	CG1537	4,27	3,44
**M9ZVK8;Q8T8V5**	La-RB;La	4,38	3,44
**Q9VZU7**	Usp5	3,96	3,43
**Q9VRR3;Q8T8U3**	CG32407	3,60	3,43
**A0A0B4LFP2;Q9W5T4**	CG12547	4,46	3,43
**Q9VTC1**	CG6418	3,33	3,43
**Q7JZD3;Q9XZ57;D3DMK3;D6W4V6**	Eb1	2,52	3,43
**A0A0B4K837;A0A0B4K6T5**	RyR	4,96	3,42
**Q9VC87**	CG18528	4,03	3,42
**M9NFH4;X2JEI9;E1JJS2;Q6IDH3;Q9VRA6;Q95TR0**	RhoGAP19D	5,89	3,42
**M9NFA7**	Ubr1	4,06	3,41

**Q8SWU4;M9PDX2**	CG4577	4,34	3,40
**L0CPX5;E1JHA4;L0CRN0;L0CR94;L0CQ56;L0CRN5;L0CRY2;L0CQ61;L0CRY8**	hrp48	2,45	3,40
**C3KGP6**	CG13908-RA	3,20	3,40
**Q9VY42**	CG1461	4,87	3,38
**A0A0B4LEK8;A1Z813;Q86NS0**	Sec24AB	3,01	3,36
**Q9VGZ3;Q9NFX2;C7LA95;O76935;B5T1X5;B5T1X0**	Irp-1B	3,81	3,36
**M9PD73;Q9VIT9;M9PDF6;M9PDR0;Q9NFV5;A0ZWM2;A0ZWN4;A0ZWN8;A0ZWN0;A0ZWM6;A0ZWM4;A0ZWP0;A0ZWN6;A0ZWM8;A0ZWN2;A0ZWN5;A0ZWM7;A0ZWM1;A0ZWM9;A0ZWM5;D0IQA2**	Tep4	5,34	3,36
**O46067**	EG:25E8,1	3,27	3,35
**A0A0B4KH10;A0A0B4KGI7;A0A0B4KGE1;A0A0B4KHS6;A0A0B4KHS7;D2NUE7**	Stat92E-RE	4,50	3,35
**E5KZ94;M9PFK7**	AGO2	2,19	3,33
**X2J5E8**	FKBP59	3,43	3,33
**M9PCN6**	numb	5,06	3,33
**E1JH79;C0PUX0;B9EQU6;A0A0B4K807;D3DN03;A0A0B4K7R5;D3DMK9**	Mlf	4,82	3,32
**A8DY82;E2QCN4;A8DY80;A8DY81;A0A0B4LEZ3;Q95TG6;C0PUX1;Q8IH39**	Not1	3,30	3,30
**Q9W3Z3;C6TP18;F6J907;Q8T0J1;Q24510**	Spat	5,73	3,29
**E1JJ98;F3YDJ2;F3YDK6**	sol	3,83	3,29
**Q9GV29**	MoxGM95	3,93	3,29
**A0A0B4LG34;A8DYM0;A8DYM1;Q9W263;Q7YU47**	Liprin-gamma;CG11206	5,26	3,29
**Q9W5R5;B5RJH6**	Slmap	1,54	3,29
**X2JF83;X2JDY2;X2JKH9;X2JF73**	HUWE1	3,86	3,28
**Q9W254;O44432**	qkr58E-2	3,18	3,27
**Q9VA54**	Rpt6R	3,57	3,26
**F3YDE2;F3YDB5**	Ald-RH	3,24	3,26
**Q8IM93;Q3ZAP5**	CG32017-RB;CG32017	1,83	3,26
**C7LAA5**	CG30122-RB	3,13	3,25

**R9Q794;Q6NL81;Q6NMY6;Q9NIU2**	RpL5;yip6	2,33	3,25
**L0MPN7;L0MPX5;Q9V4D6;L0MLN1;L0MN72;A8E766;L0MLK2;Q4V5D7**	Asator	3,03	3,25
**M9NH46;M9NDY9**	mmd	3,42	3,24
**A0A126GUN3;A0A0B4KF91**	unc-104	2,80	3,23
**A0A0B4LFL3**	Ef1beta	2,78	3,23
**Q9VT48;Q8IQD5;Q7KUD7;Q8IQD6;Q8IQD4;Q7KUD6;Q6NL70;M9PEQ4;Q8MRK3;Q86PD2;Q95SW2**	CG8177	3,94	3,22
**Q9VHE5;Q9VBH8;Q8SYG0**	RpL34	2,97	3,21
**Q9W388;Q86B59;Q7KVR7;Q494L9;Q7KVR8**	AP-1gamma	2,98	3,21
**Q7KK90**	GstE1	5,55	3,21
**M9NF47;M9PHT9;M9NFP8;M9PBX1;M9PET1;M9ND64;Q9VSM1;E1JI71;Q9VSM0;Q7YTY0;Q9VSM2;Q5BHY8**	CG43078-RA;CG32352	3,82	3,19
**Q6NP57;Q2Q3W0;A0A0B4LFK8;A1ZAW0;Q2Q3W1;Q2Q3V9;Q2Q3V7;Q2Q3V6;Q2Q3V5;Q2Q3V4;Q95YG3;Q2Q3V8**	Dcr-2	3,85	3,18
**Q8IMW9;Q9VC89;A0A0B4KGY2;Q8MR20**	CG6454	3,28	3,18
**Q0E930;A1ZB92;A1ZB93;Q6AWF2;Q9U4H2**	CG17671	1,44	3,17
**X2JCU8;Q29R20;R9PY29;Q4V5V8**	CG14126	2,15	3,17
**M9PBN7**	cpb	3,32	3,17
**Q9VF56;Q961L2**	obe	3,32	3,17
**Q9VHW4;Q960B1**	unc-45	3,60	3,17
**X2JJD6;B4YWU0;B4YWT7;B4YWU8;B4YWU7;B4YWU5;B4YWU4;B4YWU1;B4YWT9;B4YWT8;B4YWT6;A9YL27;F6J5K1;F6J5K0;F6J5J9;F6J5J7;A9YL34;A9YL31;A9YL30;A9YL28**	CG2145	1,59	3,17
**X2JDI1**	mts	2,33	3,17
**Q86BI3;Q94540;F0JAG2;Q9VUY6**	Zn72D	2,82	3,16
**X2J8Z1;X2J6P6;M9MRK1;M9MSJ0**	Pde11	2,60	3,16
**Q9XYW6;Q86DT8;Q86DT7;Q86DT5;Q86DT6**	CHIP	2,08	3,15
**Q8INW2;B7YZX0;B7FNP2;Q9VIU9;A8E6M6;Q058W1**	CG10186-RA	3,94	3,15
**Q9VWI2**	Nat1	3,71	3,14

**A0A0B4LI86**	RpII140	3,09	3,14
**E6PBV6;C9QP30**	CG1354	4,34	3,14
**A0A023T5E7**	A0A023T5E7	4,02	3,14
**I0DHK3**	Prosalpha6	3,00	3,14
**Q9W4Z0;Q7KVY9;M9PGV0;M9PGC1;C3KKB2;Q9NEH7**	PsGEF	3,98	3,12
**Q7YU20;Q9W4K1;O77290;A0A0C5K8A6;F6J363;A9YIA7;A9YIA5**	rb	4,27	3,12
**Q9Y0Y5**	epsilonCOP	2,78	3,12
**M9NE07**	CG9132	3,38	3,11
**M9PF42;Q9VLT9;Q86M30;M9NEC0;D3DML3**	Cka	2,76	3,11
**Q8T9L6**	CG13900	2,22	3,11
**A0A0B4KH09;A0A0B4K6L6;A0A0B4LHP1;A0A0B4JD02;A0A0B4JDF1;F0JAN3**	gro	3,07	3,11
**X2JE14**	Vha36-3	1,96	3,11
**E1JHJ9**	CLIP-190	3,30	3,11
**Q9VJ39;A4V0U4**	Leukotriene A(4) hydrolase	3,67	3,10
**A0A0B4JD15;Q0E9A7**	14	1,77	3,10
**A0A0B4KF69;Q9GSP5;Q0E965;G2J5W6;A1ZA05;C5WLP3;Q6NP56;C4JC89;Q95SW3**	SRPK	4,05	3,10
**M9PIA6;E1JI46;Q59E34;M9WKL1;L7S4V9;B1PGL6;B1PGM0;B1PGN0;B1PGM9;B1PGM1;B1PGK6;B1PGK4;B1PGM7;B1PGM4;B1PGM2;B1PGL8;B1PGL5;M9W9W1**	Mi-2	2,71	3,07
**Q9VJ68;Q8SY12**	CG31751	3,30	3,07
**M9PBW0**	UbcE2M	3,22	3,07
**Q8MRS5;Q9VCE7;Q95R71;O18359;D0IQG2**	KrT95D	5,65	3,06
**B7FNN6**	RpLP2-RB	2,51	3,06
**A0A0B4KFT3;A8DYG9;D3DMK5;G8HS56**	Pkc53E	2,20	3,05
**Q9VHX4**	CG2767	2,54	3,05
**A8JRH3;O46231;E1JJ21;Q9VA56;Q9VA58;C7LAH8;Q6IDG5;A0A0B4KHF2;Q9VA59;A0A0B4K7R6;A0A0B4KHW6;A0A0B4K6N4;C8VV00;C6TP61;C0PV34**	tmod	3,14	3,05

**A0A0B4LES3**	CG3107	4,16	3,05
**Q4V3L7;Q4V3F7;Q9Y112;Q4V403**	CG10863	2,71	3,05
**Q9VM19;C9QPE7;B1PHB9;B1PHB4;B1PHB3;B1PHB1;B1PHA7;B1PHA3;B1PH97;B1PH93;B1PH90;B1PH63;B1PH60;B1PH59;B1PHA8;B1PHA6;B1PH76;B1PHA2;B1PH96;B1PH95;B1PH92;B1PH91**	CG5171	4,25	3,02
**Q7KV34;Q9VYW8;F6J1N4;F6J1N0;A9YGD8;A9YGD5;F6J1N2;A9YGE4;A9YGE3;A9YGD4**	CG1561	4,82	3,02
**Q9VF51;Q8SXA6;Q86NS1**	AOX3	4,06	3,01
**H0RNL3;D3PK80;X2JES6;B5RJH2;D3PK82**	CG1552	3,18	3,01
**Q9VDC3;Q9GQM7**	AP-2sigma	2,39	3,01
**F2FB57**	tsu-RA	3,09	3,00
**Q9VNX0;Q8MZ59**	CG14562	4,17	3,00
**Q9VUC1;Q9XZT5;M9MSL3**	Hsc70Cb	5,32	3,00
**M9NEU5**	chic	5,30	2,99
**A0A0B4K657;Q6XK19;Q59DW9;Q6XK18**	btsz	2,74	2,99
**B7FNQ3**	Cg25C-RC	2,03	2,99
**A1Z7T2;A0A0B4K727;A1Z7T3;E1JH15;A0A0B4K6U3;A0A0B4LF03;A0A0B4LEI8;A0A0B4JD07;A1Z7T1;A0A0B4LFX2;A8DY76;A0A0B4LEY5;Q960L7;C8VUZ0;C8VUY9;A1Z7T4;Q7YU74**	Pkn;Pkn-RF	2,44	2,99
**E1JH01**	CSN4	2,83	2,99
**Q8T5S9;A1Z8G0**	metro	3,35	2,99
**A0A0B4LIE3;A0A0B4K7J4;A0A0B4K6A2;A0A0B4K785;A0A0B4KH06;A0A0B4K6R0;Q59DV8;C4IXY7;Q8IH40;Q8MQS5**	Gfrl	3,32	2,99
**Q9NHF8**	gag	1,63	2,98
**M9NE73**	CalpB	3,16	2,97
**Q9VWJ6;Q7YU91;X2JL73;X2JFR5**	rictor	3,30	2,96
**A8JNU6**	Nc73EF	5,92	2,96
**X2JBV9;A0A0S0WFC8;X2JG36**	Glut1	2,30	2,96
**X2J5X6;Q9VJY9;X2JAB1;Q8IP72;M9MRT5;Q4TZM6;Q27IS7;Q27IS6**	loqs	4,22	2,95

**E1JIK0**	Hrb87F	3,92	2,95
**Q9VWV6;O97355;A9UNH0;F6J873;F6J859;F6J874;F6J836;F6J843;F6J842;F6J831;F6J817**	Tsf1	3,74	2,94
**Q9VTG8;Q8SWY5**	CG7607	3,35	2,93
**E1JGX2**	Gprk1	1,65	2,93
**B5RJ67;A0A0B4KG85**	Jupiter-RD	3,59	2,92
**Q7KTW5**	CG9391	3,18	2,92
**B3STG5;B3STH6;B3STH2;B3STH0;B3STG9;B3STG7;B3STG6;B3STG4;B3STG2;B3STH3;B3STG3;A8ILB3;A8IL99;A8IL95;A8IL87;A8IL83;A8ILB0;A8ILA7;A8ILA2;I1V4Y3;A1Z6I8**	MLE;mle	4,91	2,92
**X2JE34;Q8IR13;Q9VXF8;E8NH88;Q7KUX7;Q29R50;X2JC79;Q26272**	Rbp2;Rbp2-RB	5,41	2,92
**Q9VC18;Q95RT1**	CG11089	2,63	2,91
**Q9U485;Q9VJ30;Q95TP2;Q7KT38;Q8MSQ7**	Nak	2,62	2,90
**Q9VS54;O61539;O61538;M9PEL3;Q5G1W2**	qm	2,31	2,90
**Q9VG69**	Srlp	2,79	2,90
**A0A0C4DHA1;Q2M1E6**	Cdep	3,64	2,89
**M9MRK7;X2JA38;M9MRP6;M9MRV1;X2JAP2;M9MSI8;Q8T620**	tweek	4,30	2,89
**A0A0B4LEF8**	Asap	3,76	2,88
**Q9VC06**	CG11771	3,26	2,88
**A0A0B4KHR4;A0A0B4KGH0;A0A0B4KHE2**	Dys	1,94	2,88
**Q95RY2;Q9VUQ1;Q8MQK5**	Bag2	4,81	2,88
**Q9W1Y3;A0A0B4K7E3;Q0E8Y1;Q9W1Y2;Q8T0K5;A0A0B4K7M0;F0JAJ0;D3DMX5**	PIP5K59B;PIP5K59B-RF	3,43	2,88
**Q8SXZ7;A1Z7X8**	CG12926	3,15	2,88
**A0A0B4LH64**	Atx2	2,42	2,87
**Q8IFW6;A1ZAK4;A1ZAK3;Q8T0D3;O96829**	Dek	2,84	2,87
**Q8SX89;D3DML4**	kuk;kuk-RB	2,84	2,86
**C8VV14;A4V3G1;F3YDA0;Q86NZ4**	Ald-RC;Ald;Ald-RI	6,69	2,86
**R9PY39;A8DYR5**	Shawl	3,53	2,86
**Q9VZQ9;Q8IRD5**	Strip	5,20	2,86

**M9NEW1**	Mlc-c	3,69	2,85
**Q9VTW1;X2JB05;Q9VTW0;X2JGL7;F0JAK0**	CG4300-RB	2,39	2,85
**Q9W3E2;O61732;C0HBU3;F6J4L1;A9YJZ6;A9YJZ0;F6J4K5;A9YJZ4;A9YJZ2;F6J4K8;F6J4K7;A9YK00;A9YJZ9;A9YJZ3;A9YJZ5;F6J4L0;A9YK01;F6J4L3;A9YJZ7**	PIP82	3,71	2,85
**Q5BIJ2;D2NUL2;Q0KI58;A0A0B4KHP7;B7Z0L6;A0A0B4KHB9;Q8IN90;C6SV32;Q9NFM7;B7Z0L7**	PP2A-B;PP2A-B-RK;PP2A-B-RC	5,13	2,85
**Q5LK13;L7EEU0;L7EFC0;Q5LK12;Q8SYQ3;G7H860;L7EGI4**	Dbp80;Dbp80-RB	3,12	2,85
**B6IDZ4;Q9VDP5;Q8T468**	Nlg4	4,45	2,83
**Q9VBF0;A0A0B4KH14**	CG5447	4,45	2,83
**Q9VY47;F6J2Q2;F6J2P9;A9YHP5;A9YHN7;A9YHN6;F6JA99;F6JA93;F6JA91;Q8MR65**	CG1846	4,67	2,83
**X2JBC1**	Upf1	2,49	2,83
**A0A0B4LHE7**	Vha13	4,46	2,83
**H0RNI1**	CG3267-RA	3,28	2,83
**Q9VRV1;M9PEB1;M9PEH8;Q95R81**	CG10289	2,00	2,82
**Q9VKX2;Q8MQS7**	Mdh1	2,60	2,82
**Q1EC84;Q9VNG9;Q9TY11;Q8IPP5;E1JJ52;A0A0B4KG76;A0A0B4KGK7;Q24193**	plx	3,41	2,82
**Q9VKZ8**	Usp14	3,30	2,82
**Q9VVA6**	nudC	2,42	2,81
**A4V4W0**	stnA	2,25	2,80
**Q9W0R3;Q8IRJ0;Q86PA5;Q9NHZ3;Q8WSF2**	Ptpmeg	4,48	2,78
**A0A0B4LI13**	Pkc98E	2,34	2,78
**A0A0B4JDA0;Q9VF03;Q7KPY3;Q8MSY3;Q7KND4**	mor	2,84	2,78
**Q8IQW2;Q9VWD1;C1C592**	COX6B;CG14235-RA	4,22	2,78
**X2J9Z1;D3DME3;Q6AWJ5**	svr	5,92	2,78
**M9NCX1;M9PBJ2**	l(2)gl	4,61	2,77
**Q9V9A9;E1JGY6;Q0E9N2;Q7KJC1;Q0E9N3;Q7KJB9;Q9N6X2**	l(2)01289	3,89	2,77
**Q0KHR7;Q7KUX3;C8VV45;Q960I4;Q95RN2**	Sep4;Sep4-RG	1,61	2,76

**Q8MR71**	Reg-2	2,81	2,76
**O62602;Q9U983**	lic	2,03	2,75
**Q9VUJ1;O17312;O17311**	Prosbeta2	3,02	2,75
**Q9VME1;Q6NR65**	DIP-epsilon	3,44	2,75
**Q9VPY2;Q9GUB1**	Plap	1,75	2,75
**G2J611;F6J3W3;F6J3W2;F6J3W1;F6J3W4**	wuho-RA;CG15897	1,93	2,75
**Q9VHA1;Q86P04;Q9VHA2**	SpdS	3,50	2,75
**Q9V3Y7;I7CDY4;A4VA33;A4VA32;Q6NNY4**	CG15293-RA;CG15293	2,06	2,74
**A8JUY0**	Rnp4F	3,31	2,73
**M9PB68**	poe	1,64	2,72
**Q9VP57**	pzg	1,52	2,72
**Q0E8X7**	rho	1,71	2,72
**Q9VIX7;Q8INW9;M9PDP3**	fon	4,07	2,72
**Q6NNV7;Q5LJT0;Q6IDG8;C1C3F5**	CG40485-RA	3,84	2,71
**A0A126GUS4;A0A0B4K703;A0A0B4K6D5;A0A126GUS6;A0A0B4K6N6;F0JAH5**	Mical	1,35	2,70
**Q9VUH8**	Tdrd3	3,40	2,70
**Q9V396**	CAH1	3,13	2,70
**Q9VWP8**	CG32543	3,07	2,70
**Q9V3Q9;Q9BML7;D3PFE6**	GABA-B-R1	2,55	2,70
**Q8IPA0**	Qtzl	5,69	2,69
**Q9VJQ0**	CG4168	4,50	2,69
**Q8MLS2;A9UNB7**	Rpi;CG30410	3,17	2,69
**A1Z968;Q86NR8;Q0E996**	NAT1	3,03	2,69
**Q9VRP3;Q7KMR7**	Txl	2,79	2,68
**Q59DT9;O01399;A0A0B4KI28;Q9VBJ2;O01398;O01397**	Nf1	2,97	2,68
**Q8IMF5;A0A0B4KI71**	Map205	3,11	2,68
**A0A0B4KHX3**	dco	2,84	2,67

**Q5BHU8;Q29QD2**	capu	2,38	2,67
**Q9VHH2;E1JIG3;A0A0B4KGX5;Q961J4;Q6NN53**	ird1	2,69	2,66
**Q9VSL2**	GstO3	2,09	2,66
**M9MRC9**	RpL27A	1,50	2,65
**M9NG50;M9PHG2;C7LAH9;C9QP54**	Moe;Moe-RA	3,21	2,65
**Q1EBX5**	IP15855p	2,66	2,64
**O62530**	AP-2mu	4,87	2,64
**A0A0B4LFW6;A1Z7S0;C7LAD8;Q6NP12**	Rme-8;Rme-8-RA	2,23	2,64
**X2JIQ5**	RpL17	3,44	2,63
**E1JJI5**	CkIalpha	2,45	2,63
**Q9W374;Q95U76;Q4TWT4;Q94914**	su(r)	2,65	2,63
**M9PBZ9**	eIF-4E	2,81	2,63
**Q9VR53**	wap	3,45	2,62
**Q9VGE4;Q32KE8**	GCC185;CG3532	1,81	2,62
**Q9W0D3**	CG13917	2,49	2,62
**Q9VPF3;Q961W1**	4-hydroxyphenylpyruvate dioxygenase	4,45	2,61
**Q9VHR5;N0A2N3;Q8MRT0**	CG9684-RA;CG9684	2,82	2,61
**Q7K9H6;Q960V6**	Sara	2,66	2,61
**Q9VGH5;Q8INJ6;Q8IG99**	glo	3,25	2,61
**A0A126GUN0;A0A0B4KEG4;D0IQE3**	Dgk	4,00	2,60
**Q9W2M4;Q8MR08**	CG10527	4,71	2,60
**M9PB65;M9PF26;M9PCA8;M9PCG7;M9PF31;M9PCU9;Q9VM04;M9PB64;M9PCG3;Q8MYS5**	Ziz	2,66	2,60
**Q9VH09**	CG3999	2,69	2,60
**Q9V414;Q8T080;Q95TL1**	Smg5	3,09	2,60
**Q7KK51;Q9W1Q6;Q8MLR7**	CG3530	2,92	2,60
**C4IXZ0**	Cont-RA	3,67	2,60
**I0B8M2;B6IDW5;A0A0B4KEQ1**	Hdc-RA;Hdc	4,63	2,59

**D1YSG8**	CaMKII	6,15	2,59
**Q8MT58;A0A0B4LEQ0;E8NHA0**	Cndp2	3,36	2,58
**Q7KTS3;Q9VNC9**	CG2519	1,70	2,58
**Q9VJ12;Q8MRH4**	Acn	3,32	2,58
**A0A0B4KGJ1;C7LAA7;Q7KLG8**	Sep 05	2,85	2,57
**Q9VE88;A0A0B4LIC4;C5WLU9**	CG15803	3,95	2,57
**M9PCE8**	Acer	1,82	2,57
**M9PEY6;Q95RS5**	Rat1	4,18	2,57
**M9MSK3;X2JCI2**	Src64B	2,63	2,57
**A0A0B4LFQ7**	CG9646	1,84	2,57
**Q9V9W2;Q9V9W3**	RpL6	3,71	2,56
**Q9W501**	temp	3,00	2,56
**C0MJU2**	CG11981	2,11	2,55
**M9NEC7;M9NF11;Q9W2X0**	Hk	4,44	2,55
**Q95NU8;L0CP15;L0CQQ5;L0CQQ0;L0CQP4;L0CP74;L0CQZ3;L0CP80;L0CP68;L0CP10;L0CQZ9;L0CQY4;L0CQC3;L0CQB4**	jeb	2,97	2,55
**Q9VKI8**	GH26	4,01	2,55
**Q9I7M6;C0HDP4**	Sytalpha	1,30	2,55
**Q9VS84;Q86P15;E3CTU3**	ltl;CG32372-RA	2,48	2,54
**Q960Y8**	alt	2,65	2,53
**X2JC94**	Pgam5	2,47	2,53
**Q9VYR1;Q76NR6;C6TP78**	regucalcin;regucalcin-RD	5,26	2,53
**A0A0B4LEY6**	Nacalpha	1,87	2,52
**E1JGU6;Q8MLR1;Q8IGT9;C0PDF0**	Pde8	1,80	2,52
**X2JEC6;Q8IRL5;X2JJ95;B7Z140;Q058Z7**	CG2967	2,71	2,52
**Q8MRM6;Q9W3D6**	CG12065	4,48	2,51
**A8JR81;E2QD56;A8JR82;E2QD55;E1JIT7;A8WHG2;Q8MRV5;C0PUX2;Q6IJE9**	Efa6;CG31158	3,48	2,51
**Q9NCC3**	SH3PX1	3,05	2,49

**X2JEX7**	Usp7	3,98	2,49
**E2QCS7;Q3HKQ4;C0PUX3**	CG17097	4,02	2,49
**Q9VAH7**	yata	1,74	2,49
**Q9V9V4;Q4V5F0**	CG11550	3,03	2,49
**Q9VB69;Q9U1J0;E1JIZ5;E1JIZ4**	Men-b	5,36	2,49
**A0A1B2AIV7;Q5BIL1**	RpL14	2,44	2,49
**Q9VRD6;Q6WAR9;A8JUT4**	Ntf-2	5,72	2,46
**Q9VAG5;Q6NNE1;Q8T9D9**	ca;CG31037	2,67	2,46
**B4F5L6**	CG3004	2,13	2,46
**Q9VMR6**	CG12512-RA	5,96	2,46
**A4UZI0;A0A0B4LGK5;A1ZA18;A0A0B4KFR5;A0A0B4KG24;O01349;Q6NNU4**	Khc-73	4,14	2,45
**M9PD14**	fln	1,36	2,44
**Q9VBN5**	RpL27	2,81	2,44
**Q9V3W7**	SF2	4,50	2,44
**A0A0B4KEY9;A0A0B4KFU0**	Ack-like	2,93	2,43
**Q9VV47**	CG4842	4,13	2,42
**C6TP70**	14-3-3epsilon-RA	5,58	2,42
**Q86B44;Q8IQZ1;A3FP35;A3FP36;Q1RL06;X2JCE7;Q9VX03**	GS	2,38	2,42
**A0A0B4K7Y7;A0A0B4K6K9;F9W345;F9W325;E1NZB5**	larp;larp-RB	1,61	2,41
**Q9W1H5;Q52KA4**	DCP1;CG11183	1,34	2,41
**Q8IPB7;Q9VKV1;Q9VLI0**	LManII	2,94	2,41
**Q7YU85;Q86PF3**	cindr	3,11	2,41
**A0A0B4KGX1;A0A0B4LHV4;H1UUJ8**	jar;jar-RG	5,36	2,40
**Q8SY21;A0A0B4KG94;Q9VGJ6**	CG6908	1,46	2,40
**M9PD17;Q9VW83;Q7K0T0;Q9U9B0**	gig	3,81	2,40
**Q9VMI9**	mtm	2,34	2,39
**M9PDF2**	swm	5,38	2,39

**M9PF68**	Klp68D	3,12	2,38
**C6TP77**	Edc3-RB	2,90	2,38
**A4UZL5**	GstS1	4,38	2,37
**Q9VVA4;M9PCU8**	CG9674	3,76	2,37
**Q9VRQ1;M9PHI7**	kri	3,40	2,37
**C5WLN1**	CG9940-RA	2,65	2,37
**Q9VB04;Q7KRX5;A0A0B4KH64;C8VV40;Q960S3**	btz;btz-RB	2,71	2,37
**Q9W0B8;O77285**	alphaCOP	1,79	2,37
**A0A0B4LFF8;A0A0B4K7T7;A1ZAN6;Q8T9E3;O44381;O44113;E8NH10**	RhoGEF2;RhoGEF2-RD	4,39	2,37
**Q9W1D9;A0A0B4LGH8**	Oxysterol-binding protein	2,80	2,36
**Q5U118;O18645;Q7KK41**	AMPKalpha	1,82	2,36
**Q9V9R2**	Cul2	2,34	2,36
**Q8MRV7;Q9VXQ0**	mRpL3	1,31	2,35
**E4NKG1**	Capr	3,33	2,35
**A0A0B4KEI5;E8NHA7;A0A0B4KF41**	Ars2;Ars2-RC	1,96	2,35
**B7YZQ7;A0A0B4K7G9;I0DHK6;B9EQY3**	Nurf-38;Nurf-38-RA;Nurf-38-RB	1,80	2,35
**Q9VW59**	RhoGDI	4,95	2,34
**B7YZQ1;Q9W179;Q8MLP1;Q7YZB2;Q59E61;A4UZU8;Q95U41;A8WHJ8;Q8MRH6**	slik	2,00	2,34
**Q9VRZ1;Q967S0**	Prat2	4,71	2,34
**Q7K1M4**	CG1902	3,43	2,34
**Q8IMK1;Q0KHZ4;A0A0C4DHB8;Q7K501**	CG34133	5,23	2,34
**Q9VY91;F6J2G8;F6J2G6;F6J2G4;A9YHE2;B4YX27;F6J2H2**	Pdcd4	2,24	2,33
**Q9W499;Q8MT59;C6SV02**	RpL35;RpL35-RA	1,72	2,32
**Q9V3K7;M9NF32**	BG:DS09217,4	2,79	2,32
**Q9VYB2**	CG11178	3,99	2,32
**M9PG40;Q8SYG3;D3DME9;M9PDD4**	CG40045-RA	2,20	2,32
**M9PBM2;A8JNJ9;M9PE05;M9PH77**	enc	1,30	2,31

**Q9VFP0**	CG3061	2,39	2,31
**A0A023GPM5;M9MSN3**	rdgA	1,76	2,31
**D0IQI7**	Ppn-RC	1,43	2,31
**Q9VTN9;Q8SXK3**	CG10361-RA	2,42	2,30
**M9PG76**	RpLP0	4,45	2,30
**Q9VF02;Q71V44;Q86NV8;J9XXX0;K4MNB1;Q8IG93**	Hel89B;Hel89B-RB	3,57	2,30
**E2QC90;E0R983;A0A0B4K730**	ced-6;ced-6-RC	3,66	2,30
**G4LU37**	gammaCop-RA	3,92	2,30
**M9PFH0;M9PF27;Q95SE8;F0JAQ9;Q9VU19**	Wbp2	2,96	2,30
**Q8IRE3;Q6AWD7;Q960U6;Q8IG85;W8PHN3**	gry	4,23	2,29
**Q7KS34;Q7KS35;A0A0B4K716;A8JR94;Q9VCF2;A0A0B4KHZ4;Q058V2;Q86PB4;Q95T86**	CG31140-RB;CG31140-RC	4,49	2,29
**Q8MMD3;Q8MMD2;E3CTP7**	Eps-15;Eps-15-RB	2,75	2,29
**Q7K485**	cathD	1,30	2,28
**N0D8I3;B6IDH0**	Spt6;Spt6-RA	4,05	2,28
**M9PCQ7;M9PB51;M9PCB8;M9PC63**	CG13769	2,12	2,28
**Q7JWD6;Q7KYI0**	EloC	3,36	2,28
**Q9VED8;Q7JYM9**	DNaseII	4,23	2,28
**Q9VA09**	Gycbeta100B	2,21	2,27
**M9PCE0**	Pgk	2,45	2,26
**Q9VY05**	CG9512	2,70	2,26
**Q629J6;Q24465;O18399**	pn	2,39	2,26
**Q7JVK6;Q6TMH4**	trsn	4,10	2,25
**Q86PM1;Q9VWI6**	kek5	2,11	2,25
**Q8SY53**	Ostgamma	2,30	2,25
**M9PHM6**	RpL18	4,67	2,25
**A0A0B4JD78;A0A0B4KFQ6;A0A0B4KEQ8;D3DMT3**	aPKC	4,55	2,24
**X2JFG0;Q1RKZ5;A6YH32**	r	3,62	2,24

**Q8IP94;Q8IGC5;Q9VKB0;Q8MRL7**	Aats-thr	2,97	2,24
**Q8SY36;Q8IRG9;Q8IRG8;M9PDV3;M9PE64;Q9W0E3;E8NH72**	CG12090	2,53	2,23
**Q9VJ14**	mib2	2,40	2,23
**Q7KMP8;Q29QY1**	Rpn9	1,90	2,23
**I1V4Y8**	ISWI	4,73	2,22
**Q9Y128;M9PEI8**	cert	1,99	2,22
**O44434;Q95TJ8;Q9TZN6;Q9W240;Q8T8U7;Q9W224;Q3YND6**	qkr58E-3	3,42	2,22
**Q9Y119**	Tps1	6,35	2,21
**B7Z0X3;X2JE60;Q0KHV9;X2JAH8;B7Z0W8;Q6IIN6;Q6IIN7**	rg	1,62	2,21
**A1ZBL5**	par-1	4,74	2,21
**X2JAZ3**	Dsor1	3,80	2,21
**Q9VN73**	CG1124	1,48	2,20
**Q9VW22;B7FNQ9;Q95TM6**	l(3)76BDm;CG8793-RA	2,37	2,20
**Q9VY78**	Clic	2,35	2,19
**Q9W347;M9PHH0;Q9NHX8;A9YKB9;A9YKC7;A9YKC6;A9YKC1;A9YKC0**	c12,2	3,05	2,19
**G7H829;Q8IPH6;Q5U1A3;Q8IPH7;Q7YU72;Q9VLZ3;Q9NH54;Q8IPH8**	ck	2,71	2,19
**A4V193**	pUf68	2,98	2,18
**H5V8C6**	Gycalpha99B-RA	2,85	2,18
**Q8MLS1;H9XQB1;C6TP39**	CG34424-RA	1,44	2,17
**Q9VCM6**	CG4393	3,02	2,17
**Q9VIC6;A4IJ79;Q29QD6**	Nlg3	3,77	2,17
**Q9VDY8;A0A0B4KGG5**	vib	2,86	2,17
**Q9W1Y0;A0A0B4KFY4;F3YDF1**	CG3499-RC	2,62	2,16
**M9NDW1;M9PGJ0;M9PDH5;M9PBG6;Q8MRM9**	DIP2	4,53	2,16
**O76863**	eIF2B-beta	3,38	2,16
**Q9VU92**	FOHSDR	2,71	2,16
**Q9VT15**	CG3088	1,48	2,15

**X2JGC8;X2JAR2**	Dscam2	1,85	2,15
**A1ZBH1;Q8IGB6;A8DYI9;Q7K4M9;A1A6W0**	CG15118	3,13	2,15
**M9PHV5;Q9VSV5;Q9TVG7;A0A0S0WIE7;Q32KE2**	GluRIB;Glu-RIB	2,98	2,14
**Q9GP66;Q8SXH6;D3DML0**	nahoda	3,11	2,14
**B3DMP7**	CG2827-RA	2,94	2,13
**M9PHK8**	dlg1	3,18	2,13
**Q86NM8;C4IY07**	Cdc37;Cdc37-RA	1,93	2,13
**Q2XYI2;Q9VPX5**	Vps29	2,47	2,13
**E1JHQ1**	Gs1	2,27	2,13
**M9PC10**	CG31653	3,63	2,12
**Q9VRL1;Q8IGH9;Q8IQ53**	Uev1A	4,04	2,12
**Q24474;M9PBZ2;Q9VQJ0;Q26293;A2RVE5;Q95TA9;Q26279**	Rbp9	1,49	2,12
**E1JJH5;Q1WWE7;M9PEB3;C1C536**	dlg1	2,53	2,12
**Q8WR20;Q8WR19;Q9VW45;M9PFY4**	Fibp	4,55	2,11
**A0A126GUS1;A0A126GUR3;Q9VHQ8;B5X504;A0A0B4K642;A0A0B4K609;Q9VHR2**	PQBP-1;PQBP-1-RA	1,76	2,11
**X2JGA6**	Cyp6v1	2,01	2,11
**A0A0B4KHR8;A0A0H4XWY3;A0A0B4K725;A0A0B4LHK4;A0A0B4KHZ8;U3RLS1**	mask	1,66	2,11
**Q9VR31**	Fnta	2,23	2,10
**X2JEB4;A9YJ49;A9YJ44;Q95R42**	Top3beta	3,30	2,10
**A1Z9J3**	shot	2,34	2,10
**T2FFB7;K7ZI11;Q8T9G8;K7ZI63;K7ZDS0**	Aats-ala-RB;AATS;Aats-ala	2,89	2,10
**Q7KVL7;Q9W277;Q6AWP5;Q95RP4**	Vps35	1,75	2,10
**Q9VBC4**	CG6420	3,04	2,10
**Q7K533**	Gbp2	4,70	2,10
**Q8IMX8;Q8IGW7**	LSm3	2,08	2,10
**Q59E58;Q59E59;J7JVR0;C9QPB4**	zip;zip-RC	5,71	2,10
**X2JFQ7**	eas	2,98	2,09

**Q9VGG9;Q7KSN8;Q8MQK8;Q8MSA2**	CG14730	2,97	2,09
**A8DYI2**	Ttd14	2,51	2,09
**Q9VEJ0**	Prx3	4,68	2,08
**C7LAB6**	fab1-RA	3,09	2,08
**Q9VL01;Q86PE8**	CG5390-RA;CG5390	5,24	2,08
**Q9VGF1;Q9VGF2;B9ER21**	CG12224-RA	1,82	2,08
**X2JC35**	RpL36	1,61	2,08
**Q9VKW5**	CG5355	2,51	2,08
**A0A0B4LG05**	tud	2,35	2,08
**B7YZV1;M9PDA0;M9NF02;X2BZJ7;Q8SXR0**	Pde1c	3,38	2,07
**Q9W2J5;Q8IGV8;A0A0B4LH29**	CG9480	1,96	2,07
**Q961J5**	Balat	2,13	2,07
**M9PGX7;C8VV34;Q8IGC8**	SCOT;CG1140-RB	2,65	2,07
**M9PE30;Q9VZI1**	Chd64	4,47	2,06
**Q9VIE7;Q8WSN4;C4IXY5**	bur	2,87	2,06
**Q2PDR9;Q8MR96;Q9VIJ4;Q8SXI6;Q7KT16;M9PDB2;D6W4X0;A1XXK1;A1XXK3;A1XXK2;A1XXK5;A1XXK4**	vari;vari-RB	1,58	2,06
**D2NUK9**	hoip	4,66	2,06
**Q4V565**	CG12173	1,90	2,05
**E2QC56**	PP2Cg	2,20	2,05
**F6J1D0**	sqh	2,76	2,05
**A0A0B4KEH0;Q6LEH5;Q8SWR6**	14-3-3zeta	3,19	2,04
**E1JIZ3**	CG5521	1,93	2,04
**Q27598;E2RWQ0**	Prophenoloxidase 1	5,87	2,04
**X2JFR1;Q8IR16;L7XBJ7**	nonA;nonA-RB	3,76	2,04
**Q95R98**	Protein phosphatase methylesterase 1	3,05	2,03
**A2VEF1;M9NDE4;Q9W0L8**	CG12502	2,33	2,03
**Q9VUW2;Q8MQN7**	CG17029	2,21	2,03

**M9PHG6**	Sh	2,80	2,03
**Q9W1K4;Q7YU43;P92030**	egl	3,84	2,02
**A0A0B4KEJ7;Q7JVY0;A0A0B4KEU5**	coro	1,90	2,02
**Q9VYW4;Q8MQQ7;B4YWX2;B4YWW9;A9YGF2;A9YGE9;F6J9N4;F6J9M9;B4YWX9;B4YWX8;F6J1Q4;A9YGF4;A9YGE7;F6J1P3;F6J9P5;B4YWX7;B4YWW6;F6J1Q1;F6J1P5;A9YGF0;A9YGE8;B4YWW7;B4YWW8**	CG1703	2,72	2,02
**A8E774;F6J7B3**	Cyp1;CG9916	1,39	2,02
**X2JDU0**	RpL9	3,16	2,02
**Q9W425;Q24593**	Rbcn-3A	2,70	2,02
**A4V441;Q8SY66**	sn	1,56	2,02
**Q7KXY9;Q9VM47;Q8SXU2**	Mnn1	2,51	2,01
**Q9VGM2;Q8IGA2;Q8INK3;Q8INK2**	fabp	3,41	2,01
**E1JHX5**	CG7611	1,93	2,01
**M9PHG3;X2JE67;X2JEJ9**	rdgA	2,95	2,01
**E8NH77;E6PBX0;X2JLF7;A4V4T4;F2FB48;E6PBX8;B5RJJ8;E8NH86;M9NE47;X2JCR4;F0JAG7;Q9W5W4;O96509;Q8IRZ3;X2JCQ7;Q7KWG3;T2FGB0;Q5BHW5;Q8MSA9;Q7KPH5**	sw;sw-RJ;sw-RK;Sdic3-RB;sw-RD;Sdic1;Sdic4;Sdic;Sdic3;Sdic2	2,79	2,00
**Q9V9Q4;E8NH58**	CG1416	1,91	2,00
**L0MLR4**	CaMKII	2,83	2,00
