# Supplementary material for: Blobby is a synaptic active zone assembly protein required for memory in Drosophila
Source: Nat Commun. 2025 Jan 2;16:271. doi: 10.1038/s41467-024-55382-9 (PMC11696761; doi:10.1038/s41467-024-55382-9)
Supplement: Supplementary file 2 — Reporting Summary [file 41467_2024_55382_MOESM2_ESM.pdf]

nature portfolio

Corresponding author(s): Prof. Dr. Stephan Sigrist

Last updated by author(s): Dec 6, 2024

Reporting Summary

Nature Portfolio wishes to improve the reproducibility of the work that we publish. This form provides structure for consistency and transparency in reporting. For further information on Nature Portfolio policies, see our Editorial Policies and the Editorial Policy Checklist.

Statistics

For all statistical analyses, confirm that the following items are present in the figure legend, table legend, main text, or Methods section.

n/a	Confirmed	
☐	☒	The exact sample size (n) for each experimental group/condition, given as a discrete number and unit of measurement
☐	☒	A statement on whether measurements were taken from distinct samples or whether the same sample was measured repeatedly
☐	☒	The statistical test(s) used AND whether they are one- or two-sided<br>*Only common tests should be described solely by name; describe more complex techniques in the Methods section.*
☒	☐	A description of all covariates tested
☐	☒	A description of any assumptions or corrections, such as tests of normality and adjustment for multiple comparisons
☐	☒	A full description of the statistical parameters including central tendency (e.g. means) or other basic estimates (e.g. regression coefficient) AND variation (e.g. standard deviation) or associated estimates of uncertainty (e.g. confidence intervals)
☐	☒	For null hypothesis testing, the test statistic (e.g. F, t, r) with confidence intervals, effect sizes, degrees of freedom and P value noted<br>*Give P values as exact values whenever suitable.*
☒	☐	For Bayesian analysis, information on the choice of priors and Markov chain Monte Carlo settings
☒	☐	For hierarchical and complex designs, identification of the appropriate level for tests and full reporting of outcomes
☐	☒	Estimates of effect sizes (e.g. Cohen's d, Pearson's r), indicating how they were calculated

Our web collection on statistics for biologists contains articles on many of the points above.

Software and code

Policy information about availability of computer code

Data collection	proteomics data have been deposited to the ProteomeXchange Consortium via the PRIDE partner repository: PXD058345
Data analysis	AZ segmentation analysis: available at https://github.com/ngimber/BruchpilotSegmentation

For manuscripts utilizing custom algorithms or software that are central to the research but not yet described in published literature, software must be made available to editors and reviewers. We strongly encourage code deposition in a community repository (e.g. GitHub). See the Nature Portfolio guidelines for submitting code & software for further information.

Data

Policy information about availability of data

All manuscripts must include a data availability statement. This statement should provide the following information, where applicable:

- Accession codes, unique identifiers, or web links for publicly available datasets
- A description of any restrictions on data availability
- For clinical datasets or third party data, please ensure that the statement adheres to our policy

The datasets generated during and/or analysed during the current study are available from the corresponding author on reasonable request.

Research involving human participants, their data, or biological material

Policy information about studies with human participants or human data. See also policy information about sex, gender (identity/presentation), and sexual orientation and race, ethnicity and racism.

Reporting on sex and gender	*Use the terms sex (biological attribute) and gender (shaped by social and cultural circumstances) carefully in order to avoid confusing both terms. Indicate if findings apply to only one sex or gender; describe whether sex and gender were considered in study design; whether sex and/or gender was determined based on self-reporting or assigned and methods used. Provide in the source data disaggregated sex and gender data, where this information has been collected, and if consent has been obtained for sharing of individual-level data; provide overall numbers in this Reporting Summary. Please state if this information has not been collected. Report sex- and gender-based analyses where performed, justify reasons for lack of sex- and gender-based analysis.*
Reporting on race, ethnicity, or other socially relevant groupings	*Please specify the socially constructed or socially relevant categorization variable(s) used in your manuscript and explain why they were used. Please note that such variables should not be used as proxies for other socially constructed/relevant variables (for example, race or ethnicity should not be used as a proxy for socioeconomic status). Provide clear definitions of the relevant terms used, how they were provided (by the participants/respondents, the researchers, or third parties), and the method(s) used to classify people into the different categories (e.g. self-report, census or administrative data, social media data, etc.) Please provide details about how you controlled for confounding variables in your analyses.*
Population characteristics	*Describe the covariate-relevant population characteristics of the human research participants (e.g. age, genotypic information, past and current diagnosis and treatment categories). If you filled out the behavioural & social sciences study design questions and have nothing to add here, write "See above."*
Recruitment	*Describe how participants were recruited. Outline any potential self-selection bias or other biases that may be present and how these are likely to impact results.*
Ethics oversight	*Identify the organization(s) that approved the study protocol.*

Note that full information on the approval of the study protocol must also be provided in the manuscript.

Field-specific reporting

Please select the one below that is the best fit for your research. If you are not sure, read the appropriate sections before making your selection.

☒ Life sciences ☐ Behavioural & social sciences ☐ Ecological, evolutionary & environmental sciences

For a reference copy of the document with all sections, see nature.com/documents/nr-reporting-summary-flat.pdf

Life sciences study design

All studies must disclose on these points even when the disclosure is negative.

Sample size	We used 4-6 animals per analysis, with 2-4 NMJs per animal , or 47-150 active zones depending on the experiment. These sample sizes allowed us to perform statistical analyses of sufficient confidence and were selected based on research standards and experience. for mean pixel intensity and active zone area determination (at confocal) 5 Drosophila larvae were dissected; for ephys measurments between 4-6 animals were measured; for Manders coefficient analysis (STED) 9-10 animals were analysed.
Data exclusions	- if during the e-phys measurments the measured cell/animal died were excluded from analysis
Replication	Experiments were repeated at least 2-3 times and data was reproducible.
Randomization	Randomization was not always possible. One person performed the experiment, an other person did the analysis.
Blinding	Blinding was not neccessary. All data were normalized to control situation.

Reporting for specific materials, systems and methods

We require information from authors about some types of materials, experimental systems and methods used in many studies. Here, indicate whether each material, system or method listed is relevant to your study. If you are not sure if a list item applies to your research, read the appropriate section before selecting a response.

Materials & experimental systems

n/a	Involved in the study	
☐	☒	Antibodies
☒	☐	Eukaryotic cell lines
☒	☐	Palaeontology and archaeology
☐	☒	Animals and other organisms
☒	☐	Clinical data
☒	☐	Dual use research of concern
☒	☐	Plants

Methods

n/a	Involved in the study	
☒	☐	ChIP-seq
☒	☐	Flow cytometry
☒	☐	MRI-based neuroimaging

Antibodies

Antibodies used	For more details according dilutions for each experiment, see Methods part in main manuscript:<br>mouse anti-Bruchpilot Nc82/ BRPCterm (DSHB, catalog #nc82; RRID:AB_2314866)<br>rabbit-anti BlobbyC-term (this manuscript)<br>anti-rabbit Blobbyex8b (this manuscript)<br>guinea pig-anti Unc13A (this manuscript)<br>rabbit-anti Unc13B (Bohme et al., 2016)<br>rabbit-anti GluRIID (Qin et al., 2005)<br>mouse anti-Tubulin (Sigma T9026)<br>Rabbit anti-RIM-BP (Liu et al., 2011)<br>Rabbit anti-Syd-1 (Owald et al., 2010)<br>FluoTag-X2 anti-ALFA AbberiorStar635P (N1502-Ab635P-L)<br>goat anti-HRP-Cy5 (Jackson ImmunoResearch)<br>goat anti-rabbit-Cy3 (Jackson ImmunoResearch 111-165-144)<br>goat anti-mouse or anti-rabbit Cy3 (abcam, ab97035/ ab6939)<br>goat anti-mouse or anti-guinea pig or anti-rabbit Alexa Fluor 488 (Life Technologies A11001/A11073/A11008)<br>Alexa Fluor594-coupled goat anti-rabbit (Invitrogen A32754)<br>STARRED FluoTag X2-coupled goat anti-mouse (Abberior STRED-1001-500UG)<br>anti-GFP STARRED Fluo Tag X4 (NanoTag N0304-AbRED-L)<br>goat anti-mouse ATTO490LS (Hypermol Cat.#:2109-1MG)<br>ATTO490 LS coupled goat anti-rabbit (Hypermol 2309)<br>Alexa Fluor594-coupled goat anti-guinea pig (Invitrogen 11076)<br>STARRED-coupled goat anti-mouse (Abberior STRED-1001)
Validation	If antibodies were not previously validated and published, we validated them through testing in relevant mutants through immunostainings and Western blots.

Animals and other research organisms

Policy information about studies involving animals; ARRIVE guidelines recommended for reporting animal research, and Sex and Gender in Research

Laboratory animals	Third-instar larvae or adult Drosophila melanogaster were used in this study.<br>The following strains were used: w1118 , blobbyNull, blobbyGFP, blobby-KDRT-4xSTOP, blobby-ALFA, cacGFP , cacGFP; blobbyNull, brpNull, Mef-2-Gal4, Ok6-Gal4, KD-recombinase, blobbygRNA, Gal80ts,Cas9/+;VT30559/+, Gal80ts,Cas9/blobbygRNA;VT30559/+
Wild animals	NA
Reporting on sex	For all xperiments, both male and female third instar larvae were used.
Field-collected samples	All animals for experiments were kept constantly in 25°C incubators with dark/light cycle.
Ethics oversight	Ethical approval was not required

Note that full information on the approval of the study protocol must also be provided in the manuscript.

Plants

Seed stocks

Report on the source of all seed stocks or other plant material used. If applicable, state the seed stock centre and catalogue number. If plant specimens were collected from the field, describe the collection location, date and sampling procedures.

Novel plant genotypes

Describe the methods by which all novel plant genotypes were produced. This includes those generated by transgenic approaches, gene editing, chemical/radiation-based mutagenesis and hybridization. For transgenic lines, describe the transformation method, the number of independent lines analyzed and the generation upon which experiments were performed. For gene-edited lines, describe the editor used, the endogenous sequence targeted for editing, the targeting guide RNA sequence (if applicable) and how the editor was applied.

Authentication

Describe any authentication procedures for each seed stock used or novel genotype generated. Describe any experiments used to assess the effect of a mutation and, where applicable, how potential secondary effects (e.g. second site T-DNA insertions, mosiacism, off-target gene editing) were examined.
